# Mitochondria-derived H_2_O_2_ triggers liver regeneration via FoxO3a signaling pathway after partial hepatectomy in mice

**DOI:** 10.1038/s41419-023-05744-w

**Published:** 2023-03-28

**Authors:** Hua Bai, Cong-Wen Fang, Ying Shi, Song Zhai, An Jiang, Ying-Na Li, Lin Wang, Qi-Ling Liu, Geng-Yao Zhou, Jia-Hao Cao, Jia Li, Xue-Kang Yang, Xu-Jun Qin

**Affiliations:** 1 Frontiers Science Center for Flexible Electronics, Xi’an Institute of Flexible Electronics (IFE) and Xi’an Institute of Biomedical Materials & Engineering, Northwestern Polytechnical University, Xi’an, 710072 China; 2 Department of Nutrition and Food Hygiene, School of Preventive Medicine, The Fourth Military Medical University, Xi’an, 710032 China; 3 School of Public Health, Shaanxi University of Chinese Medicine, Xianyang, 712046 China; 4 Weiyang District Center for Disease Control and Prevention, Xi’an, 710016 China; 5 Department of Infectious Diseases, The Second Affiliated Hospital, Xi’an Jiaotong University, Xi’an, 710004 China; 6 Department of General Surgery Ward 4, The Second Affiliated Hospital, Xi’an Jiaotong University, Xi’an, 710004 China; 7 People’s Hospital of Zhuhai High-tech Industrial Development Zone, Zhuhai, 519085 China; 8 Department of Hepatobiliary Surgery, Xijing Hospital, The Fourth Military Medical University, Xi’an, 710032 China; 9 Key Laboratory of Aerospace Medicine of Ministry of Education, School of Aerospace Medicine, The Fourth Military Medical University, Xi’an, 710032 China; 10 Department of Burns and Cutaneous Surgery, Xijing Hospital, The Fourth Military Medical University, Xi’an, 710032 China; 11 Key Laboratory for Space Biosciences and Biotechnology, School of Life Sciences, Northwestern Polytechnical University, Xi’an, 710072 China

**Keywords:** Liver diseases, Preclinical research, Mitochondria

## Abstract

Reactive oxygen species (ROS) can induce oxidative injury and are generally regarded as toxic byproducts, although they are increasingly recognized for their signaling functions. Increased ROS often accompanies liver regeneration (LR) after liver injuries, however, their role in LR and the underlying mechanism remains unclear. Here, by employing a mouse LR model of partial hepatectomy (PHx), we found that PHx induced rapid increases of mitochondrial hydrogen peroxide (H_2_O_2_) and intracellular H_2_O_2_ at an early stage, using a mitochondria-specific probe. Scavenging mitochondrial H_2_O_2_ in mice with liver-specific overexpression of mitochondria-targeted catalase (mCAT) decreased intracellular H_2_O_2_ and compromised LR, while NADPH oxidases (NOXs) inhibition did not affect intracellular H_2_O_2_ or LR, indicating that mitochondria-derived H_2_O_2_ played an essential role in LR after PHx. Furthermore, pharmacological activation of FoxO3a impaired the H_2_O_2_-triggered LR, while liver-specific knockdown of FoxO3a by CRISPR-Cas9 technology almost abolished the inhibition of LR by overexpression of mCAT, demonstrating that FoxO3a signaling pathway mediated mitochondria-derived H_2_O_2_ triggered LR after PHx. Our findings uncover the beneficial roles of mitochondrial H_2_O_2_ and the redox-regulated underlying mechanisms during LR, which shed light on potential therapeutic interventions for LR-related liver injury. Importantly, these findings also indicate that improper antioxidative intervention might impair LR and delay the recovery of LR-related diseases in clinics.

## Introduction

The liver is a vital metabolic organ with the powerful capability of regeneration, which is essential for maintaining liver function in response to acute or chronic injuries [1, 2]. During the process of liver regeneration (LR), the proliferation of liver cells, particularly hepatocytes, significantly contributes to the recovery of the original size and mass. These normally quiescent cells can be activated to enter the cell cycle in response to liver injuries such as two-thirds partial hepatectomy (PHx) [3, 4]. Although multiple growth factors, inflammatory cytokines, transcription factors, and signaling pathways have been reported to be involved in LR [3–5], the mechanisms of regulation on cell proliferation after PHx remain to be elucidated.

Reactive oxygen species (ROS), a family of active molecule metabolites of oxygen, can induce oxidative injury and has been regarded only as toxic cellular “waste products” for many decades [6]. ROS-induced cellular damage and inflammation are well-documented in the pathogenesis of many liver diseases [7]. Recently ROS have been recognized as crucial molecular regulators of cell signaling and functions, including cell proliferation [8–10]. Studies have reported a significant increase of lipid oxidation as early as 6 hours after PHx in rats [11, 12], suggesting the overproduction of ROS occurred after PHx. To date, the definite role of ROS in LR remains unclear.

Mitochondria are the primary source of ROS and responsible for more than 90% of ROS production under normal conditions [13, 14]. PHx has been demonstrated to induce significant changes in mitochondrial ultrastructure, permeability as well as mitochondrial respiratory function [15, 16]. The PHx-induced impairment of mitochondrial oxidative phosphorylation is accompanied by the increased oxidation of mitochondrial proteins and the decreased levels of mitochondrial antioxidants, such as glutathione and glutathione peroxidase [15, 17, 18], indicating the overproduction of mitochondrial ROS (mtROS) after PHx. However, little is known about the exact nature of mtROS and its role in LR after PHx. In our previous study, we found that hydrogen peroxide (H_2_O_2_) level was correlated to the transition from quiescence to proliferation in hepatocytes, suggesting the involvement of H_2_O_2_ in LR [19]. We speculate that H_2_O_2_ derived from mitochondria may be beneficial in triggering the LR after PHx.

FoxO3a, a member of the forkhead box O (FoxO) family, is a redox-regulated transcription factor involved in diverse cellular processes, including proliferation, apoptosis, metastasis, redox homeostasis, cell metabolism, aging, and cancer biology [20–22]. FoxO3a has been proposed as a sensor for redox signaling [23]. ROS can regulate FoxO3a protein levels at transcriptional and post-transcriptional levels, including phosphorylation, acetylation, methylation, and ubiquitination, by many different upstream redox-sensitive signaling cascades [24, 25]. On the other hand, FoxO3a has been shown to play a critical role in the regulation of cell proliferation by inducing cell cycle arrest through transcription of multiple cell cycle kinase inhibitors (CKI), and the best-described CKI downstream of FoxO3a is p27 [26, 27]. Thus we hypothesized that the redox-sensitive FoxO3a might be the critical node in the signaling pathways mediating the ROS-triggered LR after PHx.

In the present study, by employing the mitochondria-targeted ROS probes and antioxidants, as well as the mice with liver-specific overexpression of mitochondria-targeted catalase (mCAT) or liver-specific knockdown of FoxO3a, we demonstrated that mitochondria-derived H_2_O_2_ triggered LR via FoxO3a signaling pathway. Our findings provided the initial evidence of the beneficial and critical role of mitochondrial H_2_O_2_ and the redox-regulated underlying mechanism during LR, which laid the foundation for potential therapeutic intervention targets for LR and related diseases. Importantly, these findings also indicate that improper antioxidative intervention might impair LR and delay the recovery of LR-related diseases in clinics.

## Results

### MtROS correlated with the cell proliferation during LR after PHx

**Fig. 1:**
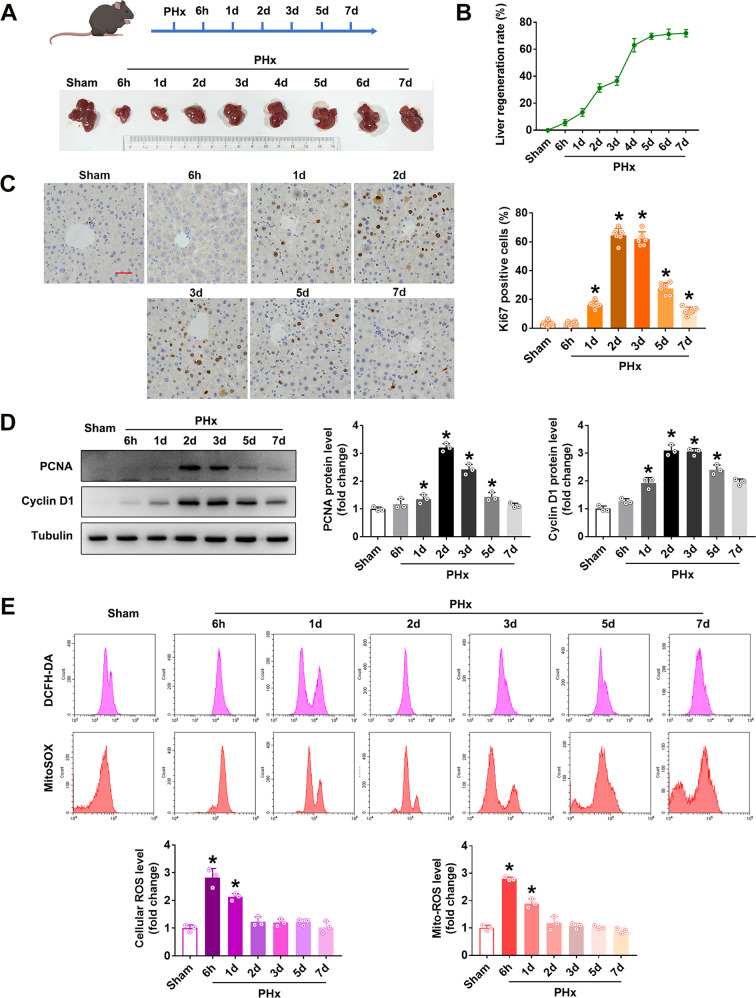
Time course of liver regeneration (LR) and reactive oxygen species (ROS) production after 70% partial hepatectomy (PHx) in mice. Liver samples were taken, and LR and ROS production was determined at different time points (6 h, 1 d, 2 d, 3 d, 5 d, and 7 d) after the C57 mice were subjected to PHx. **A** Schematic representation of the experimental procedure and representative macroscopic images of remnant liver. **B** LR rate, *n* = 6. **C** Immunohistochemistry staining and quantification of Ki67, bar = 50 µm, *n* = 6. **D** Western blot analysis and quantification of PCNA and Cyclin D1 (three independent experiments). **E** Flow cytometry assay and quantification of cellular ROS (probed with DCFH-DA) and mitochondrial ROS (probed with MitoSOX) (three independent experiments). Data are shown as means ± SEM. *P < 0.05 vs Sham group.

The liver starts regeneration immediately after PHx. The LR rate at different time points showed that the liver gained weight steadily with a sharp increase from the 2nd to 4th days, then slowed down on the 5th day and almost recovered the weight by the 7th day after PHx (Figs. 1A, B), confirming the regeneration pattern in the previous report [30]. As cell proliferation significantly contributes to the LR, we assessed the proliferation marker Ki67 by immunohistochemistry. The positive rates of Ki67 increased steadily and reached the peak at around the 2nd day after PHx and then went down to the quiescent level (Fig. 1C), which was validated by the protein levels of other proliferation markers and also the key cell cycle proteins PCNA and Cyclin D1 (Fig. 1D). These data suggested that the cell proliferation-mediated LR may be completed within one week, with the peak cell proliferation activity at around the 2nd day after PHx. Therefore, we evaluated cell proliferation on the 2nd day and LR rate on the 4th day after PHx in the following experiments.

As ROS has been reported as an essential stimulator for cell proliferation and ROS level changes with the cell cycle in a regular fluctuation manner [9, 31, 32], we isolated the primary hepatocytes to measure the intracellular ROS by DCFH-DA staining. The fluorescence of DCFH-DA increased significantly at 6 hours after PHx and then decreased gradually, demonstrating the increase of ROS level at the early stage of LR (Fig. 1E). Given that mitochondria are the primary source of ROS, we also detected the mtROS by MitoSOX staining at the same time. The mtROS increased simultaneously with the increase in intracellular ROS, suggesting that mitochondria-derived ROS may be responsible for the increased ROS after PHx (Fig. 1E). These results suggest that the increase of mitochondria-derived ROS in the early stage may contribute to PHx-induced ROS as well as cell proliferation during LR.

### Inhibition of mtROS remarkably suppressed LR after PHx

**Fig. 2:**
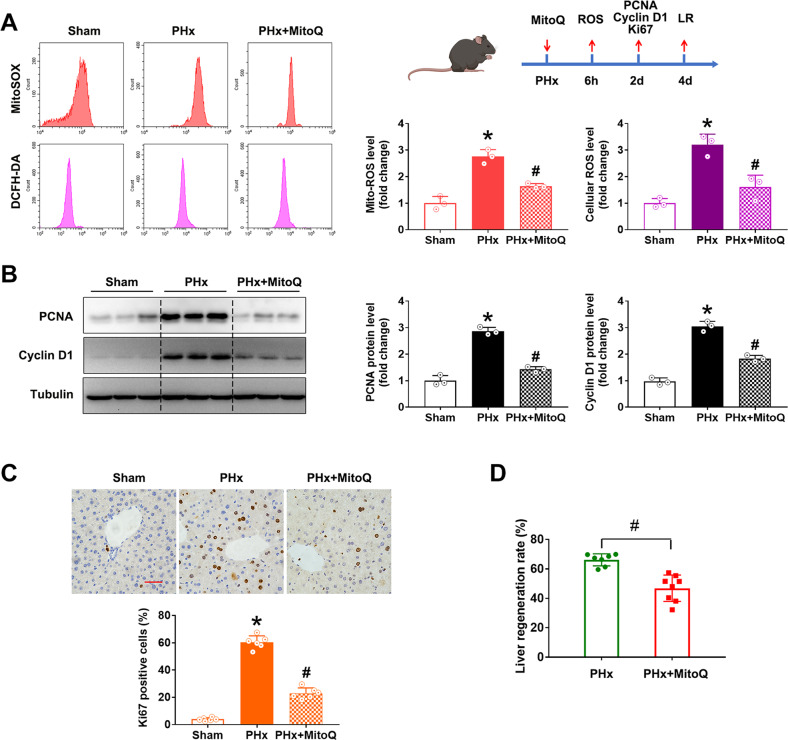
The mitochondrial-targeted antioxidant MitoQ inhibited LR after PHx in mice. The C57 mice were intraperitoneally injected with MitoQ (2 mg/kg BW) immediately after PHx and followed once daily. The ROS production was detected at 6 hours, and LR was determined on the 2th day after PHx. **A** Schematic representation of the experimental procedure and flow cytometry assay and quantification of cellular ROS (probed with DCFH-DA) and mitochondrial ROS (probed with MitoSOX) (three independent experiments). **B** Western blot analysis and quantification of PCNA and Cyclin D1 (three independent experiments). **C** Immunohistochemistry staining and quantification of Ki67, bar = 50 µm, *n* = 6. **D** LR rate, *n* = 7. Data are shown as means ± SEM. *P < 0.05 vs Sham group and ^#^*P* < 0.05 vs PHx group.

In order to evaluate the role of increased ROS on LR as well as the mtROS on the intracellular ROS level, we employed a mitochondria-targeted antioxidant, Mitoquinone (MitoQ), to block the mtROS. As expected, MitoQ treatment significantly attenuated the mtROS level after PHx. Similarly, the intracellular ROS level was also blocked by MitoQ (Fig. 2A), demonstrating the critical role of mtROS in ROS production after PHx. Meanwhile, MitoQ treatment significantly reduced the cell cycle protein levels of PCNA and Cyclin D1 (Fig. 2B) as well as the proliferation marker Ki67 positive rate (Fig. 2C), suggesting the inhibition of cell proliferation by mitochondria-targeted antioxidants. Furthermore, scavenging mtROS by MitoQ significantly inhibited the LR rate (Fig. 2D). Together, all these results suggested that mitochondria-derived ROS promoted cell proliferation and LR after PHx.

### Mitochondria-derived H_2_O_2_ triggered LR after PHx

**Fig. 3:**
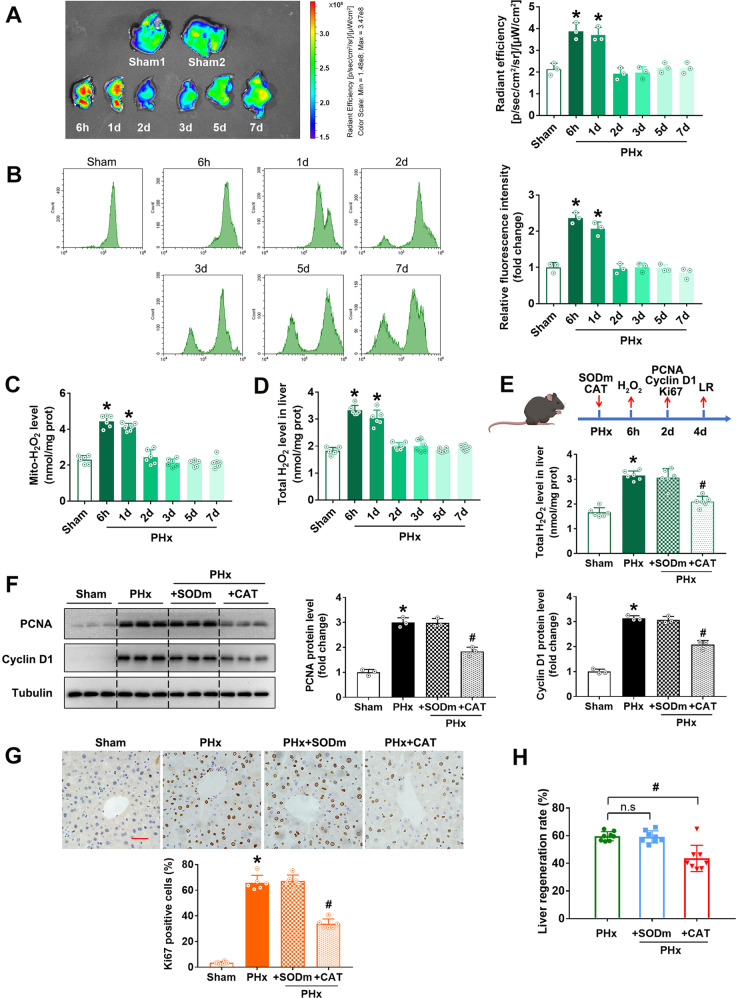
Mitochondria-derived H_2_O_2_ promoted LR after PHx in mice. **A**–**D** The mitochondrial H_2_O_2_ and total H_2_O_2_ were determined at different time points (6 h, 1 d, 2 d, 3 d, 5 d, and 7 d) after the C57 mice were subjected to PHx. **A** Representative liver fluorescence images and quantified data of radiant efficiency of Mito-LX probe (three independent experiments). **B** Flow cytometry assay and quantification of mitochondrial H_2_O_2_ probed with Mito-LX (three independent experiments). **C** and **D** Mitochondrial H_2_O_2_ and total H_2_O_2_ in liver tissue assessed by Amplex red kit, *n* = 6. **E**–**H** The C57 mice were intraperitoneally injected with O_2_^•−^ scavenger SOD mimic (5 mg/kg BW) or H_2_O_2_ scavenger CAT (10 mg/kg BW) immediately after PHx and followed by once every day. The liver H_2_O_2_ was detected at 6 hours and LR was determined on the 2nd day after PHx. **E** Schematic representation of the experimental procedure and total H_2_O_2_ in liver tissue detected by Amplex red kit, *n* = 6. **F** Western blot analysis and quantification of PCNA and Cyclin D1 (three independent experiments). **G** Immunohistochemistry staining and quantification of Ki67, bar = 50 µm, *n* = 6. (H) LR rate, *n* = 7. Data are shown as means ± SEM. *P < 0.05 vs Sham group and ^#^*P* < 0.05 vs PHx group.

Under physiological conditions, ROS are initially produced in mitochondria as superoxide, which is subsequently catalyzed and converted to H_2_O_2_ immediately by superoxide dismutases (SODs) [9]. H_2_O_2_ has a much longer half-life and can exist for a long time in cells. H_2_O_2_ is the key molecule to mediate redox signals [33, 34]. In the current study, we employed a novel mitochondria-targeted H_2_O_2_-specific probe (Mito-LX) with long waves to enable visualization of the mitochondrial H_2_O_2_ signal in vivo [28]. The H_2_O_2_-specific fluorescence signal of the liver imaging increased rapidly after PHx and then downregulated slowly (Fig. 3A), which was further confirmed in the isolated primary hepatocytes by flow cytometry (Fig. 3B), supporting the stimulation of mitochondria-derived H_2_O_2_ by PHx. Further, we also employed a commercial Amplex red H_2_O_2_ quantification kit to verify the H_2_O_2_ production of mitochondria. Consistent with the data of Mito-LX, mitochondrial H_2_O_2_ levels increased significantly at the early stage and then returned to sham levels after PHx (Fig. 3C). We also found that the profile of the total H_2_O_2_ level of liver tissues (Fig. 3D) coincided with that of the mitochondrial H_2_O_2_ level. Moreover, MitoQ treatment inhibited mitochondrial H_2_O_2_ after PHx (Supplementary Fig. 1). Overall, the results above suggested that mitochondria were the primary source of ROS, and H_2_O_2_ might be the critical messenger of ROS to regulate LR after PHx.

In order to validate the essential role of H_2_O_2_ in the LR after PHx, we used a SOD mimic (SODm), Mn(III)TMPyP, to scavenge superoxide and catalase (CAT) to scavenge H_2_O_2_. As expected, SODm did not affect the H_2_O_2_ levels, while CAT significantly reduced the total H_2_O_2_ level in liver tissues (Fig. 3E). Next, we evaluated the effect of scavenging H_2_O_2_ on cell proliferation. CAT treatment significantly reduced proliferation markers PCNA and Cyclin D1 protein levels (Fig. 3F) and Ki67 positive rate (Fig. 3G), while SODm did not show any effect, indicating that scavenging H_2_O_2_ could abolish the cell proliferation induced by PHx. This inhibition of cell proliferation by H_2_O_2_ scavenger did attenuate LR (Fig. 3H). All these results suggested that mitochondria-derived H_2_O_2_ is necessary for LR after PHx.

Mitophagy is the specific autophagic elimination of mitochondria to regulate mitochondrial number [35]. In order to further confirm the essential role of mitochondria-derived H_2_O_2_ in the LR after PHx, we employed a mitophagy inducer urolithin A (UA) to regulate the mitochondria number to regulate the mitochondria-derived H_2_O_2_ level [36]. PHx decreased protein levels of mitophagy markers Parkin, PINK1, Nix, and FUNDC1 (Supplementary Fig. 2), indicating the inhibition of mitophagy. UA treatment significantly restored these PHx-inhibited protein levels of Parkin, PINK1, Nix, and FUNDC1 (Supplementary Fig. 3A), suggesting mitophagy elevation. Mtphagy Dye (CAS 2137473-96-0) is a specific probe for mitophagy detection, as it can yield strong fluorescence when the autophagosomes containing mitochondria fuse with lysosomes [37]. The fluorescence of Mtphagy Dye deceased after PHx while UA treatment remarkably increased the fluorescence of Mtphagy Dye, demonstrating the improvement of UA on the inhibited mitophagy after PHx (Supplementary Figs. 3B, C). The enhancement of mitophagy by UA was further validated by the decreased mtDNA copy number (Supplementary Fig. 3D). More importantly, UA treatment significantly inhibited PHx-induced mitochondria H_2_O_2_ level as well as the total H_2_O_2_ level in the liver (Supplementary Figs. 3E, F), with the inhibitions on the protein levels of PCNA, Cyclin D1 (Supplementary Fig. 3G), the positive rate of Ki67 (Supplementary Fig. 3H), and the LR (Supplementary Fig. 3I). These results demonstrated that the reduction of mitochondria-derived H_2_O_2_ by induction of mitophagy inhibited LR after PHx, which provided more evidence of the essential role of mitochondria-derived H_2_O_2_ in LR after PHx and suggested regulating mitophagy to be a potential intervention strategy.

Besides mitochondria, NADPH oxidases (NOXs) family is another important source of ROS production, and the critical role of NOXs in ROS-related diseases has been documented in the literature [38]. Only three out of seven NOXs family members (NOX1-NOX7), NOX1, NOX2, and NOX4, have been reported to be detected in liver tissue [39, 40]. In the present study, we did not detect any change in NOX2 protein level, with a moderate reduction in the protein levels of NOX1 and NOX4 at the early stage of LR after PHx (Supplementary Fig. 4A). In order to investigate the involvement of NOXs in ROS/H_2_O_2_ production and LR after PHx, a widely used NOXs inhibitor, Apocynin with two different dosages, was used to inhibit NOXs activities (Supplementary Fig. 4B). We found that Apocynin treatments did not affect PHx-induced increased H_2_O_2_ production (Supplementary Figs. 4C, D), suggesting PHx-induced ROS/H_2_O_2_ was independent of NOXs. Furthermore, Apocynin treatments did not affect the levels of proliferation markers PCNA, Cyclin D1 and Ki67, or the LR rate (Supplementary Fig. 4E–G) after PHx. These results demonstrated that NOXs did not participate in the ROS-regulated LR after PHx.

### FoxO3a signal pathway was involved in the process of LR after PHx

**Fig. 4:**
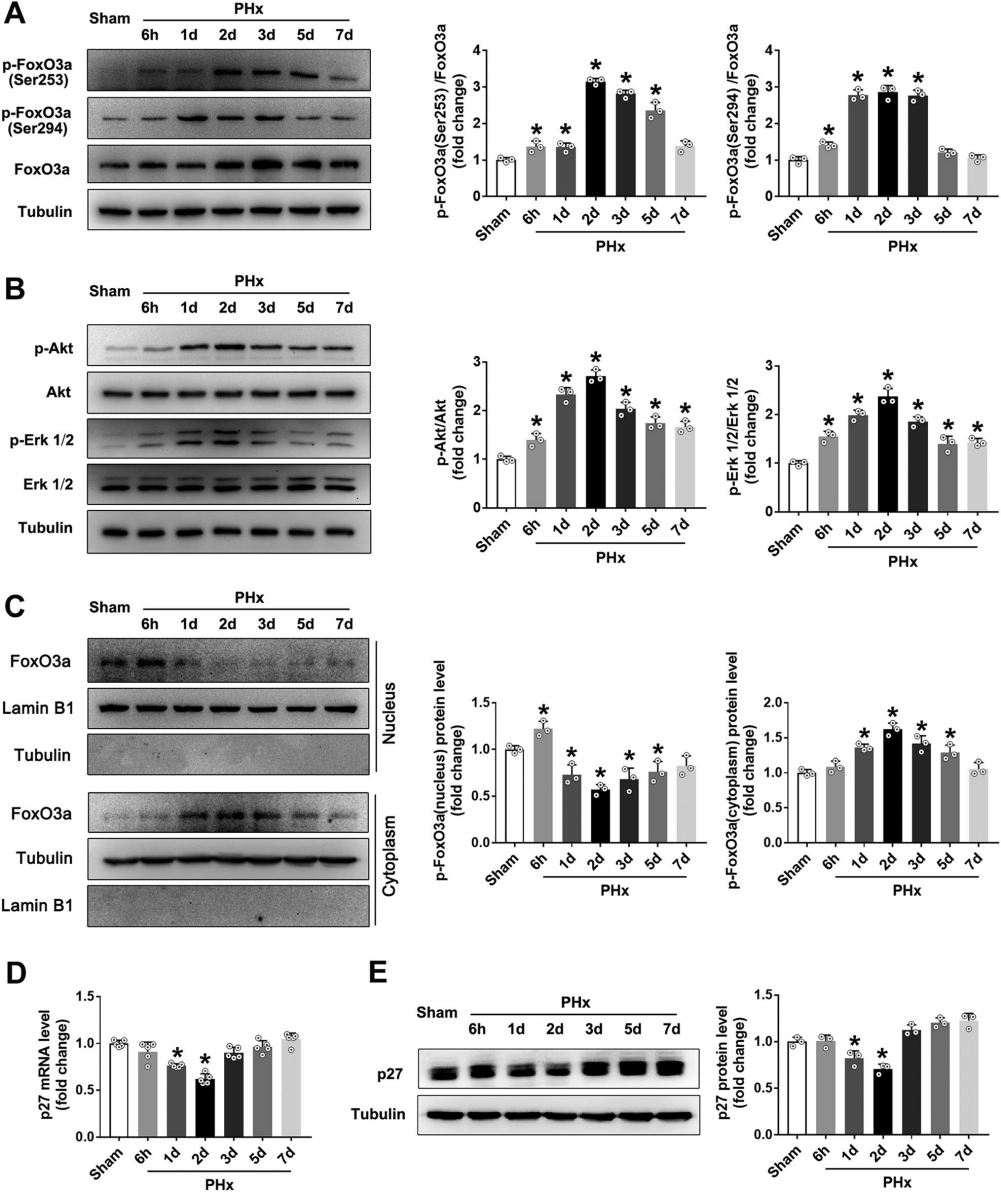
The changes of FoxO3a signaling pathway at different time points after PHx in mice. After the C57 mice were subjected to PHx, the FoxO3a signaling pathway in the liver was determined at different time points (6 h, 1 d, 2 d, 3 d, 5 d, and 7 d). **A** Western blot analysis and quantification of FoxO3a and its phosphorylation forms (Ser253 and Ser294). **B** Western blot analysis and quantification of Akt and Erk with their phosphorylation forms. **C** Western blot analysis and quantification of FoxO3a protein levels in nucleus and cytoplasm. **D** Q-PCR determination of p27 expression, *n* = 5. **E** Western blot analysis and quantification of p27 protein level. Data are shown as means ± SEM of three independent experiments. *P < 0.05 vs Sham group.

To deal with the increased ROS, the antioxidant defense system is usually activated to protect from severe oxidative damage. In the present study, surprisingly, the expressions of many important antioxidants (SOD1, SOD2, CAT, GPx1, Prx1, Prx3 and Trx1) decreased significantly after PHx (Supplementary Fig. 5). Nrf2 and FoxO3a are the most important upstream antioxidative transcription factors in regulating the expressions of antioxidants. However, Nrf2 protein level increased, accompanied by the elevated expressions of its transcriptional target proteins (HO-1 and HQO-1) (Supplementary Fig. 6), indicating the activation of Nrf2. The decreased expressions of the antioxidants (Supplementary Fig. 5) strongly suggested that FoxO3a was the key antioxidative transcription factor impaired after PHx, as it has been reported to directly regulate the expressions of SOD2, CAT, GPx1, and Prxs [41, 42]. FoxO3a phosphorylation is one of the most important post-translational modifications to regulate its transcription function [20, 21]. Firstly, we measured the total protein level of FoxO3a and its phosphorylation forms at Ser253 and Ser294. We found that phosphorylation forms of FoxO3a at Ser253 and Ser294 increased significantly and peaked around the 2nd day after PHx, with a modest increase in the total FoxO3a protein level (Fig. 4A). Given phosphorylation of FoxO3a at Ser253 is mainly mediated by Akt while phosphorylation of FoxO3a at Ser294 is mainly mediated by Erk, we further detected the Akt and Erk phosphorylation. Both Akt and Erk were phosphorylated and activated immediately after PHx, and their phosphorylation profiles were consistent with those of phosphorylation forms of FoxO3a at Ser253 and Ser294 (Fig. 4B), supporting the phosphorylation regulation of FoxO3a by Akt and Erk. It has been reported that phosphorylation of FoxO3a at Ser253 and Ser294 induces its translocation from the nucleus to the cytoplasm, resulting in the loss of its transcription activity [20, 21]. To confirm this, we measured the FoxO3a protein levels in nucleus and cytoplasm fractions (Fig. 4C). The decrease of nuclear FoxO3a level, and the increase of cytoplasmic FoxO3a level supported the translocation of FoxO3a from the nucleus to the cytoplasm after PHx. Protein p27, a crucial cell cycle inhibitor, is a direct transcriptional target downstream of FoxO3a, by which FoxO3a shows potent regulation of cell proliferation [26, 27]. We found that both the mRNA level and the protein level of p27 decreased simultaneously with the nuclear export of FoxO3a (Figs. 4D, E), indicating that FoxO3a transcription function was impaired after PHx. All these results above suggested that Akt/Erk/FoxO3a/p27 pathway might be involved in the LR after PHx.

### FoxO3a mediated LR triggered by mitochondria-derived H_2_O_2_ after PHx

**Fig. 5:**
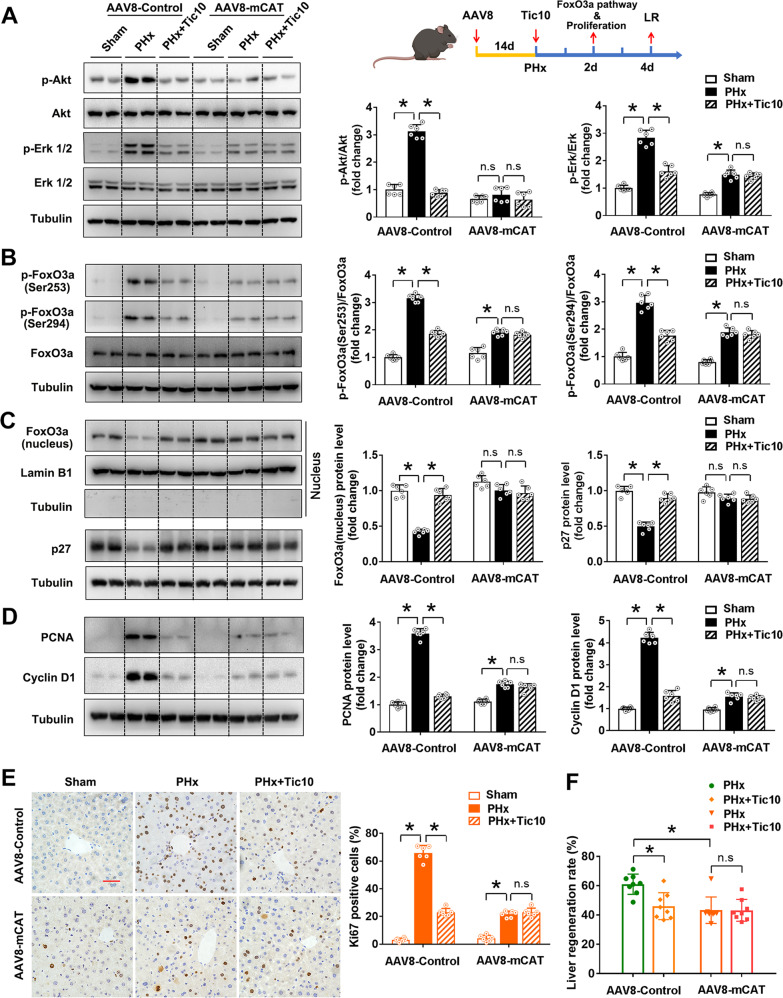
FoxO3a pathway activation attenuated mitochondria-derived H_2_O_2_ triggered LR after PHx in mice. The C57 mice were injected with a recombinant AAV8 carrying CAT gene with mitochondria-targeted sequence to overexpress mitochondria-targeted CAT (mCAT) specifically in the liver. The mice were intraperitoneally injected with Akt and Erk inhibitor Tic10 (25 mg/kg BW) immediately after PHx and followed once daily. The Akt/Erk/FoxO3a/p27 pathway and LR were determined on the 2nd day after the mice were subjected to PHx. **A** Schematic representation of the experimental procedure, western blot analysis, and quantification of Akt and Erk with their phosphorylation forms (three independent experiments). **B** Western blot analysis and quantification of FoxO3a and its phosphorylation forms (Ser253 and Ser294) (three independent experiments). **C** Western blot analysis and quantification of FoxO3a protein level in the nucleus and its target p27 protein level (three independent experiments). **D** Western blot analysis and quantification of PCNA and Cyclin D1 (three independent experiments). **E** Immunohistochemistry staining and quantification of Ki67, bar = 50 µm, *n* = 6. **F** LR rate, *n* = 8. Data are shown as means ± SEM. Significance was designated with *P < 0.05.

In order to convincingly demonstrate the mitochondria-derived H_2_O_2_ promotes LR via Akt/Erk/FoxO3a/p27 pathway, we employed mice with overexpression of mitochondria-targeted CAT (mCAT) in the liver (Supplementary Fig. 7A). We found that overexpression of mCAT almost abolished the PHx-induced activation of Akt and Erk, phosphorylation of FoxO3a at Ser253 and Ser294, translocation of FoxO3a from the nucleus to the cytoplasm, decreased expression of targeted protein p27, as well as the increased cell proliferation (illustrated by the markers Cyclin D1, PCNA and Ki67) and LR rate (Fig. 5), further demonstrating the essential role of mitochondria-derived H_2_O_2_ in the LR after PHx. In addition, we tested a well-characterized inhibitor, Tic10, which could inhibit both Akt and Erk and lead to the activation of FoxO3a [43]. In the present study, Tic10 treatment also efficiently inhibited PHx-induced Akt/Erk/FoxO3a/p27 pathway, cell proliferation, and LR rate, indicating the critical role of Akt/Erk/FoxO3a/p27 pathway in LR after PHx. More importantly, the combination of mCAT overexpression with Tic10 treatment did not result in additive inhibition on Akt/Erk/FoxO3a/p27 pathway, cell proliferation, or LR rate (Fig. 5), suggesting that mitochondria-derived H_2_O_2_ promoted PHx-induced LR mainly through Akt/Erk/FoxO3a/p27 pathway.

**Fig. 6:**
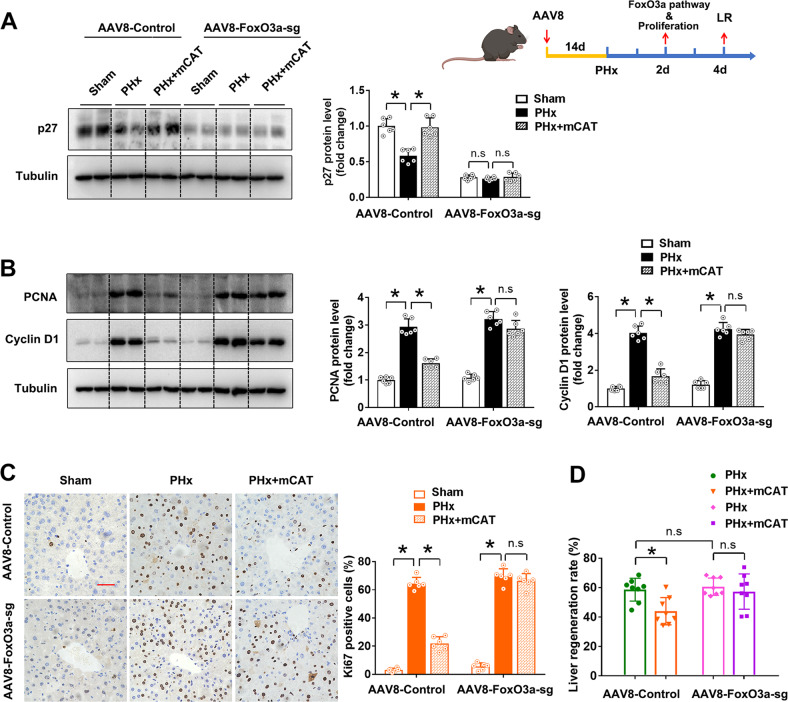
FoxO3a knockdown ameliorated the LR inhibition by overexpression of mCAT after PHx in mice. The C57 mice were injected with a recombinant AAV8 carrying mitochondria-targeted CAT (mCAT) gene and/or a recombinant AAV8 carrying the sgRNA sequence for FoxO3a to overexpress mCAT and/or knockdown FoxO3a specifically in the liver. The LR were determined on the 2nd day after the mice were subjected to PHx. **A** Schematic representation of the experimental procedure and western blot analysis and quantification of p27 protein level (three independent experiments). **B** Western blot analysis and quantification of PCNA and Cyclin D1 (three independent experiments). **C** Immunohistochemistry staining and quantification of Ki67, bar = 50 µm, *n* = 6. **D** LR rate, *n* = 8. Data are shown as means ± SEM. Significance was designated with *P < 0.05.

In order to confirm the critical role of FoxO3a in LR after PHx, we depleted FoxO3a protein specifically in the liver by AAV8-sgRNA mediated CRISPR-Cas9 technology (Supplementary Fig. 7B). We found, in the mice injected with AAV8 with empty vector, PHx significantly decreased of protein level of p27, and increased protein levels of proliferation markers PCNA, Cyclin D1, and Ki67 as well as the LR rate, while overexpression of mCAT effectively restored the expression of p27, inhibited cell proliferation and LR rate. However, in the mice with hepatocyte-specific knockdown of FoxO3a by AAV8-sgRNA, all of the alterations on cell proliferation and LR induced by overexpression of mCAT were almost abolished (Fig. 6). These results demonstrated that FoxO3a played an essential role in mediating the mitochondria-derived H_2_O_2_-triggered LR process after PHx.

## Discussion

In the past few decades, LR has received extensive studies as understanding the underlying mechanisms will benefit the treatment of liver diseases in clinics. Despite the significant progress in unraveling the molecular mechanisms of LR, the initiation events of LR are not fully understood. ROS, which has long been regarded as the toxic by-products of metabolism, has been reported in the literatures, but most previous studies focus on adverse effects of ROS on LR. In the present study, we demonstrated the beneficial role of mitochondria-derived H_2_O_2_ in triggering LR after PHx. Moreover, we elucidated that mitochondria-derived H_2_O_2_ induced translocation of FoxO3a from the nucleus to the cytoplasm by activating the upstream kinases Akt and Erk, resulting in the reduction of cell cycle kinase inhibitor p27, and finally triggering LR after PHx.

ROS-induced oxidative stress and damage have been well-documented as a driver of cancer, diabetes, cardiovascular diseases, neurodegenerative diseases, and liver diseases [44, 45]. In contrast, the physiological functions of ROS received much less attention and is not fully understood. Recently, ROS have been established as critical signaling messengers in a variety of cellular processes, including cell proliferation [8, 9, 46]. Studies have shown that the cell cycle is a redox cycle, with an increase in intracellular ROS levels during the progression from G1 to S to G2 and M phases. ROS are demonstrated to be critical regulators of quiescent cells’ entry into the cell cycle [31, 41]. In previous studies, ROS production and lipid peroxidation have been reported in a number of species after PHx, including mice, rats, and pigs [11, 12, 47]. Although α-tocopherol treatment inhibits rat LR [48], overexpression of antioxidant enzymes SOD and GPx1 does not affect the LR [49]. Moreover, enhancing ROS level by knockout nuclear factor E2-related factor 2 (Nrf2), an essential antioxidant transcription factor, or uncoupling protein-2 (UCP2) impairs LR after PHx [50, 51]. In other LR models induced by APAP-/CCL_4_-intoxicated mice, lack of mitochondrial topoisomerase I increase oxidative injury, and inhibiting oxidative stress promotes LR [52, 53]. In our present study, the ROS level increased significantly at an early stage, and scavenging ROS by chemical antioxidant or overexpression endogenous antioxidant effectively inhibited cell proliferation and LR after PHx, revealing the essential and beneficial role of ROS in LR. The contradictory findings of our study with previous studies may be due to the double-edged sword role of ROS, which shows beneficial effects under physiological conditions or toxic effects under pathological conditions depending on the dosage of ROS.

ROS are generated from many intracellular sources, including mitochondria, NADPH oxidases, endoplasmic reticulum, cytochrome P450, monoamine oxidase, xanthine oxidase, cyclooxygenase, glycolate oxidase, hydroxyacid oxidase, aldehyde oxidase, and amino acid oxidase [10, 54]. Even though mitochondria are believed to be the major source of ROS production under normal conditions [13, 14], little is known about the role of mtROS in the LR induced by PHx. In the present study, by employing the mitochondria-targeted antioxidants, we found that scavenging mtROS could significantly suppress cellular ROS, cell proliferation, and LR, demonstrating the key role of mtROS in LR after PHx. As we know, ROS are generated from the leakage of electrons in mitochondrial oxidative phosphorylation (OXPHOS). The electrons leaked from respiratory chains are transported to molecular oxygen to form superoxide union (O_2_^•−^), which will be converted to H_2_O_2_ by SODs rapidly. H_2_O_2_ is further catalyzed to H_2_O and O_2_ by CAT [9, 55, 56]. Even H_2_O_2_ can further induce hydroxyl radical (^•^OH) production by the Fenton reaction or other kinds of ROS, O_2_^•−^ and H_2_O_2_ are the two major kinds of ROS in the mitochondria [54, 57]. O_2_^•−^ is an active free radical with a half-life of about 10^−6^ sec at 37 °C, while H_2_O_2_, as a two-electron oxidant, is much more stable. Moreover, the overall cellular concentration of O_2_^•−^ is maintained at about 10^–11^ M, much lower than that of H_2_O_2_, at 10^–8^ M [58]. H_2_O_2_ has been demonstrated to use water channels to cross cell membrane rapidly, and is recognized as the major ROS in redox regulation of biological activities [10, 33, 34]. Our previous study showed that H_2_O_2_ level was correlated to the transition from quiescence to proliferation in hepatocytes [19]. In the present study, by using the mitochondria-specific probe for H_2_O_2_ and the quantitative kit, we displayed direct evidence of mitochondrial H_2_O_2_ increase at the early stage of LR after PHx. Reduction of mitochondrial H_2_O_2_ level directly by overexpression of mitochondria-targeted H_2_O_2_ scavenger mCAT or indirectly by induction of mitophagy effectively inhibited LR, demonstrating the essential role of mtH_2_O_2_ in triggering the LR after PHx. This might explain why overexpression of antioxidant enzymes SOD and GPx1 does not affect the LR in a previous study [49], as SOD catalyzes O_2_^•−^ to produce H_2_O_2_ while GPx1 scavenging H_2_O_2_ needs to consume GSH, which also been found in decline after PHx [59]. Moreover, by intervention on the activity of NOXs, the other important source of ROS, we demonstrated that NOXs were not involved in the PHx-induced ROS production, providing additional evidence to support the essential role of mitochondria-derived H_2_O_2_.

The remaining question is how mitochondria-derived H_2_O_2_ regulates LR after PHx. Nrf2 and FoxO3a are the two most crucial transcription factors to sense and respond to redox signaling. In the present study, the data of Nrf2 and its transcription targets (HO-1, NQO1, GPx4 and SOD3) suggested the activation of Nrf2 after PHx, which is consistent with the previous reports [60–62]. However, the role of Nrf2 in LR remains controversial, as constitutive activation Nrf2 impaired LR [60], while pharmacological activation of Nrf2 enhanced the restoration of liver volume and improved liver function [61]. Nevertheless, Nrf2 is a key transcription factor of antioxidants, and deletion of Nrf2 impairs LR by enhancing oxidative stress [50], suggesting the critical role of Nrf2 activation in maintaining redox homeostasis in LR. Notably, even Nrf2 was activated in the present study. We found that the expressions of some key antioxidants (SOD1, SOD2, CAT, GPx1, Prx1, Prx3 and Trx1) as its targets in the downstream decreased after PHx, suggesting the other important transcriptional factor for these antioxidants in the upstream, FoxO3a, was impaired after PHx. In a previous study, the deceased expression of FoxO3a is observed at the early stage of LR after PHx due to the direct transcriptional regulation by p53 and p73 [63]. A recent study reports that FoxO3a negatively controls hepatocyte proliferation and FoxO3a deletion accelerates LR [64]. Taken together, FoxO3a is a redox-sensitive transcription factor and a vital regulator of cell proliferation [21, 23], making it the best candidate to bridge mitochondria-derived H_2_O_2_ with LR after PHx. Even ROS have been implicated in regulating FoxO3a at multiple levels, post-translational modifications, especially phosphorylation, are the most common ways to modulate FoxO3a functions [23–25]. Akt and Erk are two well-established upstream kinases, which phosphorylate FoxO3a and induce its translocation from the nucleus to the cytoplasm, thus suppressing its transcription function [21, 22]. We previously reported the activation of the Erk pathway [19], while the others reported the activation of Akt pathway during the process of LR after PHx [65], with an additional study showing that Akt regulated LR through inhibition of FoxO1 [66]. In the present study, we found PHx-induced H_2_O_2_ increased the phosphorylation of FoxO3a at the specific sites by activating both Akt and Erk, thus reducing transcription and expression of its target p27 in the downstream, a key cell cycle inhibitor, leading to the cell proliferation and LR. Further activation of FoxO3a impaired the H_2_O_2_-triggered LR while knockdown of FoxO3a almost abolished the inhibition of LR by overexpression of mCAT, demonstrating that FoxO3a mediated mitochondria-derived H_2_O_2_ triggered LR after PHx. These findings not only unraveled the mechanism of mitochondria-derived H_2_O_2_ regulating LR, but also provided an important potential target for LR.

There were still several limitations remaining in our present study. Firstly, even though our findings provided the initial evidence of the beneficial role of ROS, especially mitochondria-derived H_2_O_2_ in LR, whether the drugs targeting mitochondria to produce more H_2_O_2_ further would accelerate LR remained to be tested. In previous studies, high levels of ROS-induced oxidative damage and cell death have been reported to impair LR [50, 51]. The threshold of this switch from beneficial effect to toxic effect needs further study in the future. Secondly, as we know, the regulation of FoxO3a is much more complex. We only elucidated the phosphorylations of FoxO3a by both Akt and Erk, resulting in its nuclear exportation and transcriptional inhibition. We did not further find out if activation Akt or Erk alone would be sufficient to inhibit FoxO3a/p27 pathway, nor did we explore the roles of all of the upstream regulators. Thirdly, as the primary source of ROS and H_2_O_2_, we did not evaluate the mitochondrial function after PHx, or in the intervention studies. Elucidating these remaining questions in the future will significantly improve the potential application of targeting mitochondria-derived H_2_O_2_ in therapeutical translation.

**Fig. 7:**
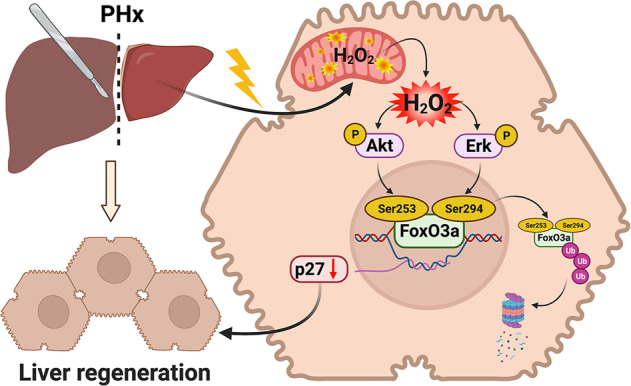
Schematic representation of mitochondria-derived H_2_O_2_ triggering liver regeneration via FoxO3a signaling pathway after partial hepatectomy in mice. After PHx, the mitochondria-derived H_2_O_2_ increased the phosphorylation of FoxO3a at the specific sites (Ser253 and Ser294) by activating both Akt and Erk, thus reducing transcription and expression of its target p27 in the downstream, a key cell cycle inhibitor, leading to the cell proliferation and LR. (This artwork was created at http://BioRender.com).

In conclusion, even though PHx-induced ROS have been reported for a long time, the role of ROS and the underlying mechanisms still need to be fully understood. Our present study demonstrates that mitochondria-derived ROS are essential in triggering the LR after PHx. Particularly, mitochondria-derived H_2_O_2_ promoted LR through Akt/Erk/FoxO3a/p27 signaling pathway (a schematic diagram of this study is shown in Fig. 7). Our findings uncover the beneficial role of mitochondrial H_2_O_2_ and the redox-regulated underlying mechanisms during LR, which sheds light on potential therapeutic interventions for LR-related liver injury. Importantly, these findings also indicate proper level of ROS in certain liver diseases or liver surgeries may be beneficial to liver recovery. Improper antioxidative intervention should be avoided as it may impair LR and delay the recovery of LR-related diseases in clinics.

## Materials and methods

### Animals and reagents

Male C57BL/6 mice (8–10 weeks old) were obtained from the Experimental Animal Research Center of the Fourth Military Medical University (Xi’an, China). Based on our preliminary experiments, cohorts of mice were randomized into different treatment groups. The mice with overexpression of mitochondria-targeted catalase (mCAT) were generated by injection (tail vein) of a recombinant adeno-associated viral vector serotype 8 (AAV8) expressing mouse CAT with a mitochondrial-targeting sequence (AAV8-mCAT) (Hanbio, Shanghai, China). To knockdown FoxO3a specifically in the liver, a recombinant AAV8 carrying the sgRNA sequence for FoxO3a (5′-GTCTTCATCGTCGTCCTCCT-3′) was injected through the tail vein into tamoxifen-inducible hepatocyte-specific Cas9 expression mice, which were generated through crossing between Alb-CreERT2 (tamoxifen-inducible hepatocyte-specific Cre under the control of mouse albumin regulatory elements, B6.129S-Alb^tm1.1(CreERT2)Smoc^) and Rosa26-CAG-LNL-Cas9 knock-in mice (C57BL/6-Gt(ROSA)26Sor^tm1(CAG-LNL-Cas9)Smoc^) from Shanghai Model Organisms Center (SMOC). The animals were maintained in a 12-hour light-dark cycle and allowed access to water and diet *ad libitum*. All animal experiments were approved by the Northwestern Polytechnical University Animal Ethics Committee and performed following the Guide for the Care and Use of Laboratory Animals (8th edition, 2011). The stock solutions of MitoQ (0.4 mg/ml), SOD mimic (Mn(III)TMPyP) (1 mg/ml), and CAT (2 mg/ml) were prepared with 0.9% normal saline. Apocynin was dissolved in DMSO and then diluted with 0.9% normal saline to get the stock solution (1 and 10 mg/ml).

### Partial hepatectomy

The mice were subjected to 70% PH, as we previously described [19]. Briefly, the mice were anesthetized with isoflurane, and a vertical incision just below the sternum was made to expose the liver. 70% PH was performed by ligating and removing the large left lateral lobe and two median lobes of the liver. For the sham operation, the mice were anesthetized and entered the abdominal cavity by incision without excision of the liver. The mice were anesthetized to collect liver tissues or to isolate primary hepatocytes at the indicated time after PHx for further experimental analysis.

### Immunohistochemistry assay

Liver tissues were fixed in 4% buffered polyoxymethylene immediately after harvesting. Then paraffin-embedded liver sections were stained with the Ki67 antibody. The staining was visualized, and the images were acquired with a light microscope (Olympus, Tokyo, Japan). The rates of positive staining cells were calculated in 5 randomly selected fields of the staining sections in each group.

### Isolation of primary hepatocytes and detection of intracellular and mitochondrial ROS/H_2_O_2_ by flow cytometry

Primary hepatocytes were isolated by two-step in situ collagenase perfusion as described previously with modification [19]. Mice were anesthetized with isoflurane. The liver was perfused in situ via the portal with Krebs Ringer Buffer (7.25 g sodium chloride, 1 g D-glucose, 2.1 g sodium bicarbonate, 0.5 g potassium chloride, 4.75 g HEPES per liter) and 1.7 mM ethylenediaminetetraacetic acid (KRB + EDTA) for less than 10 min, while the inferior vena cava was used as outflow. The perfusion was continued with a digestion buffer (50 ml KRB buffer containing 45 mg type IV collagen and 3 ml of 125 mM CaCl_2_) for 10 min. After digestion, the liver was removed from the animal and gently broken to release the cells in a cold KRB buffer. Afterward, the cell suspension was filtered through gauze (70 µm) and centrifuged for 5 min at 50 × *g* at 4 °C. Two additional washing steps with KRB buffer were performed. Then a volume of 4 ml of cold Percoll® solution (9 parts Percoll® to 1 part 1.5 M NaCl, pH 5-5.5) was used for every 6 ml of cell suspension (5 million cells/ml) and centrifuged (100 g, 4 °C, 10 min). The procedure produced approximately 10^7^ cells/g of the liver. The freshly separated hepatocytes were directly used for ROS and H_2_O_2_ detection.

Fluorescence probes, 2′,7′-dichlorodihydrofluorescein diacetate (DCFH-DA) (Sigma-Aldrich, St. Louis, MO) and MitoSOX (Invitrogen, Carlsbad, CA) were used to detect intracellular and mitochondrial ROS as previously described [19]. A novel fluorescence probe Mito-LX reported by our group, was used to measure mitochondrial H_2_O_2_ levels [28]. Briefly, one million cells were incubated with 10 μM probe in 1 ml PBS at 37 °C for 30 min in the dark. After centrifugation at 100 × *g* for 5 min, the pellets were resuspended in 200 μl PBS. Fluorescence measurements were carried out using a Beckman CytoFlex S flow cytometer (Beckman, Brea, CA) at channels for DCFH-DA (λ_ex_ = 488 nm, λ_em_ = 525 nm), MitoSOX (λ_ex_ = 488 nm, λ_em_ = 585 nm), and Mito-LX (λ_ex_ = 405 nm, λ_em_ = 610 nm) respectively.

### Mitochondrial H_2_O_2_ detection by Mito-LX liver imaging and Amplex red kit

The probe Mito-LX has a long emission wavelength, making it possible to view the H_2_O_2_-specific fluorescence signal of the whole liver directly. 3 hours before the fluorescence imaging, the mice were given Mito-LX via intravenous tail injections (10 μM Mito-LX, 100 μl/20 g BW). 3 hours later, the mice were sacrificed, and the livers were removed to be imaged, and the H_2_O_2_ specific fluorescence signal was quantified by IVIS imaging system (PerkinElmer, Waltham, MA).

The mitochondria of liver tissue were isolated by a Mitochondria Extraction kit (Keygen Biotech, China). The H_2_O_2_ levels in mitochondria as well as in the liver tissue were quantitatively detected by an Amplex red kit (Invitrogen, Carlsbad, CA).

### Western blot analysis

Liver tissues were lysed with a homogenizer in RIPA buffer with protease and phosphatase inhibitors, and the total proteins were extracted by centrifugation. Nuclear and cytoplasmic fractions were isolated by NE-PER Nuclear and Cytoplasmic Extraction Reagents (Thermo Fisher Scientific, Waltham, MA). After quantified with a BCA protein assay kit (Thermo Fisher Scientific), equal amounts of proteins were separated by sodium dodecyl sulfate polyacrylamide gel electrophoresis (SDS-PAGE) and electro-transferred onto polyvinylidene fluoride (PVDF) membranes. After blocking for 2 h with 5% skimmed milk, the membranes were incubated with indicated primary antibodies overnight at 4°C. The primary antibodies were visualized using horseradish peroxidase-conjugated anti-mouse or anti-rabbit secondary antibodies and developed by an enhanced chemiluminescence kit (Millipore, St. Louis, MO). The protein band signals were visualized and quantified with a Quantity One System (Bio-Rad, Hercules, CA). Tubulin or Lamin B1 was used as the loading control for total or nuclear proteins, respectively. A detailed list of primary antibodies was provided in Supplementary Table 1.

### Real-time PCR

Total RNA from liver tissue was isolated with Trizol (Invitrogen, Carlsbad, CA), and the concentration was measured by Nanodrop 2000 (Thermo Fisher Scientific, Waltham, MA). Reverse transcription of RNA was performed with iScript™ cDNA synthesis kit (Bio-Rad, Hercules, CA) and subsequent quantitative real-time PCR with Power SYBR™ Green PCR Master Mix (Life Technologies, Carlsbad, CA) on the CFX Connect real-time system (Bio-Rad, Hercules, CA). All of the samples were amplified with β-actin as an endogenous loading control. The relative expressions of the genes were calculated by 2^-∆∆CT^ method. A detailed list of PCR primers was provided in Supplementary Table 2.

### MtDNA copy number

For mtDNA copy number measurement, we followed the procedure as previously described [29]. Liver DNA was extracted using the Universal Genomic DNA Purification Mini Spin Kit (Beyotime, Shanghai, China). One primer pair specific for the mtDNA (16S rRNA) and another specific for the nuclear DNA (Hexokinase 2, HK2), were designed for relative quantification for mtDNA copy number. The primer sequences for the mitochondrial 16S rRNA gene were as follows: forward primer, 5′-CCGCAAGGGAAAGATGAAAGAC-3 ′; reverse primer, 5′-TCGTTTGGTTTCGGGGTTTC-3′. The primer pair used for the amplification of the nuclear gene HK2 was as follows: forward primer, 5′-GCCAGCCTCTCCTGATTTTAGTGT-3′; reverse primer, 5′-GGGAACACAAAAGACCTCTTCTGG-3′. mtDNA copy number was measured using a real time quantitative polymerase chain reaction (PCR) using a CFX connect real-time system (BioRad). A comparison of 16S rRNA DNA expression relative to HK DNA expression will give a measure of mtDNA copy number to nDNA copy number ratio.

### Liver regeneration rate

The rate of regenerating liver after PHx was calculated by the following equation: [C-(B-A)]/B*100%. A is the resected liver weight. B is the estimated total liver weight at the time of resection, which is calculated as A divided by 70%. C is the weight of the liver at the time of sacrifice.

### Statistical analysis

Data are presented as means ± SEM. For the data of intervention studies by chemical reagents, one-way ANOVA was employed, while for the data of time course study after PHx and gene-manipulation studies (mCAT overexpression and FoxO3a knockdown by AAV), two-way ANOVA was employed, followed by a post-hoc test with Bonferroni. For the qualification data of western blot, flow cytometry and liver imaging with small samples (three independent experiments), a nonparametric test, Kruskal–Wallis test was used. Statistical significance was accepted at *P* < 0.05.

## Supplementary information

Supplemental Data

Original Data File

Checklist-CDDIS-22-3463

## Data Availability

The supporting data are available from the corresponding authors on reasonable request.
