# Supplementary material for: Mitochondria-derived H2O2 triggers liver regeneration via FoxO3a signaling pathway after partial hepatectomy in mice
Source: Cell Death Dis. 2023 Mar 28;14(3):216. doi: 10.1038/s41419-023-05744-w (PMC10050396; doi:10.1038/s41419-023-05744-w)
Supplement: Supplementary file 1 — Supplemental Data [file 41419_2023_5744_MOESM1_ESM.pdf]

Supplemental Materials

S-Fig 1

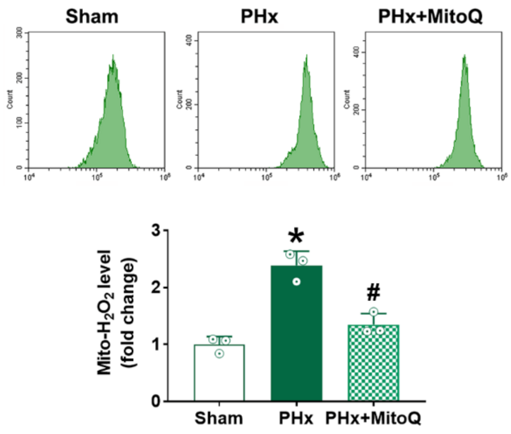

S-Fig 1: The mitochondrial targeted antioxidant MitoQ inhibited mitochondrial H_2O_2 level after PHx in mice. The C57 mice were intraperitoneal injected with MitoQ (2mg/kg BW) immediately after the mice were subjected to PHx. 6 hours later, the mitochondrial H_2O_2 was analyzed by flow cytometry with Mito-LX as the probe. Data are shown as means ± SEM of three independent experiments. *$p < 0.05$ vs Sham group and #$p < 0.05$ vs PHx group.

S-Fig 2

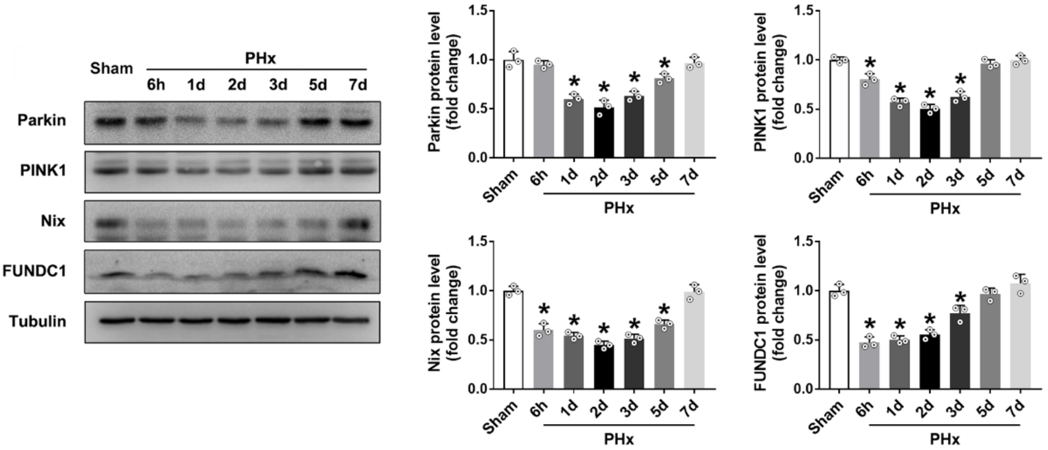

S-Fig 2: The changes of mitophagy markers at different time points after PHx in mice. After the C57 mice were subjected to PHx, the protein levels of mitophagy markers Parkin, PINK1, Nix and FUNDC1 in the liver was determined at different time points (6 h, 1 d, 2 d, 3 d, 5 d and 7 d) by Western blot analysis. Data are shown as means ± SEM of three independent experiments. *$p < 0.05$ vs Sham group.

S-Fig 3

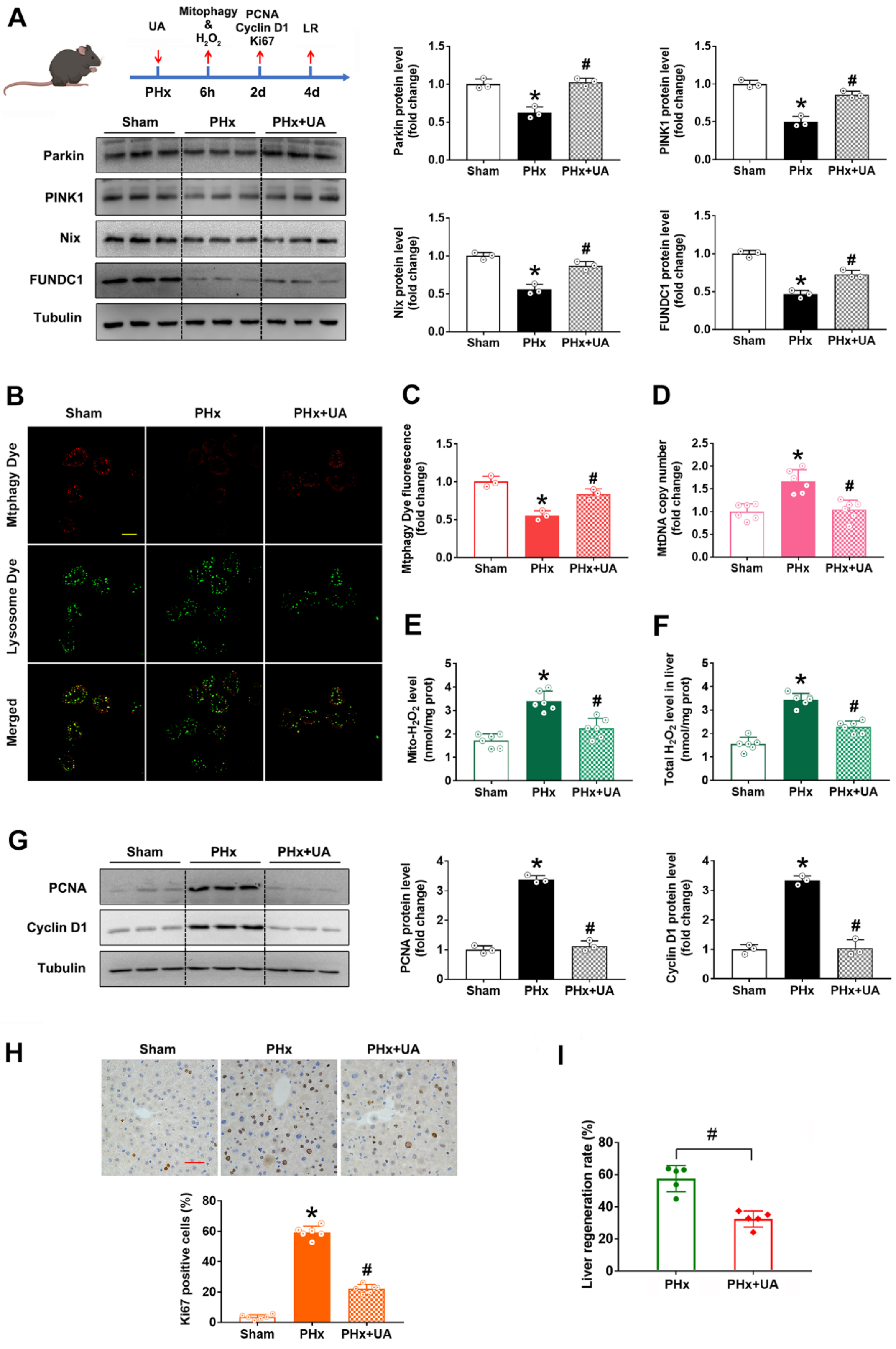
A
UA
Mitophagy & H_2O_2
PCNA Cyclin D1 Ki67
LR
PHx
6h
2d
4d
Sham
PHx
PHx+UA
Parkin
PINK1
Nix
FUNDC1
Tubulin
Parkin protein level (fold change)
PINK1 protein level (fold change)
Nix protein level (fold change)
FUNDC1 protein level (fold change)
B
Mtphagy Dye
Lysosome Dye
Merged
C
Mtphagy Dye fluorescence (fold change)
D
MtDNA copy number (fold change)
E
Mito-H_2O_2 level (nmol/mg prot)
F
Total H_2O_2 level in liver (nmol/mg prot)
G
PCNA
Cyclin D1
PCNA protein level (fold change)
Cyclin D1 protein level (fold change)
H
Ki67 positive cells (%)
I
Liver regeneration rate (%)

S-Fig 3: Inhibition of mitochondrial H_2O_2 by mitophagy attenuated LR after PHx in mice. The mice were intraperitoneal injected with mitophagy inducer UA (5mg/kg BW) immediately after PHx and followed by once every day. The H_2O_2 production was detected at 6 hours, the mitophagy markers (Parkin, PINK1, Nix and FUNDC1), mtDNA copy number and LR was determined at the 2th day after PHx. (A) Schematic representation of the experimental procedure and western blot analysis and quantification of the mitophagy markers induced by UA after PHx (three independent experiments). (B) Confocal images showing mitophagy by Mtphagy Dye staining (Dojindo, Cat#MD01), bar = 20 μm. (C) Flow cytometry quantification of Mtphagy Dye fluorescence, n=3. (D) Q-PCR determination of mtDNA copy number, n=6. (E) and (F) Mitochondrial H_2O_2 and total H_2O_2 detected by Amplex red kit, n=6. (G) Western blot analysis and quantification of PCNA and Cyclin D1 (three independent experiments). (H) Immunohistochemistry staining and quantification of Ki67, bar = 50 μm, n=6. (I) LR rate, n=5. Data are shown as means ± SEM. *$p < 0.05$ vs Sham group and #$p < 0.05$ vs PHx group.

S-Fig 4

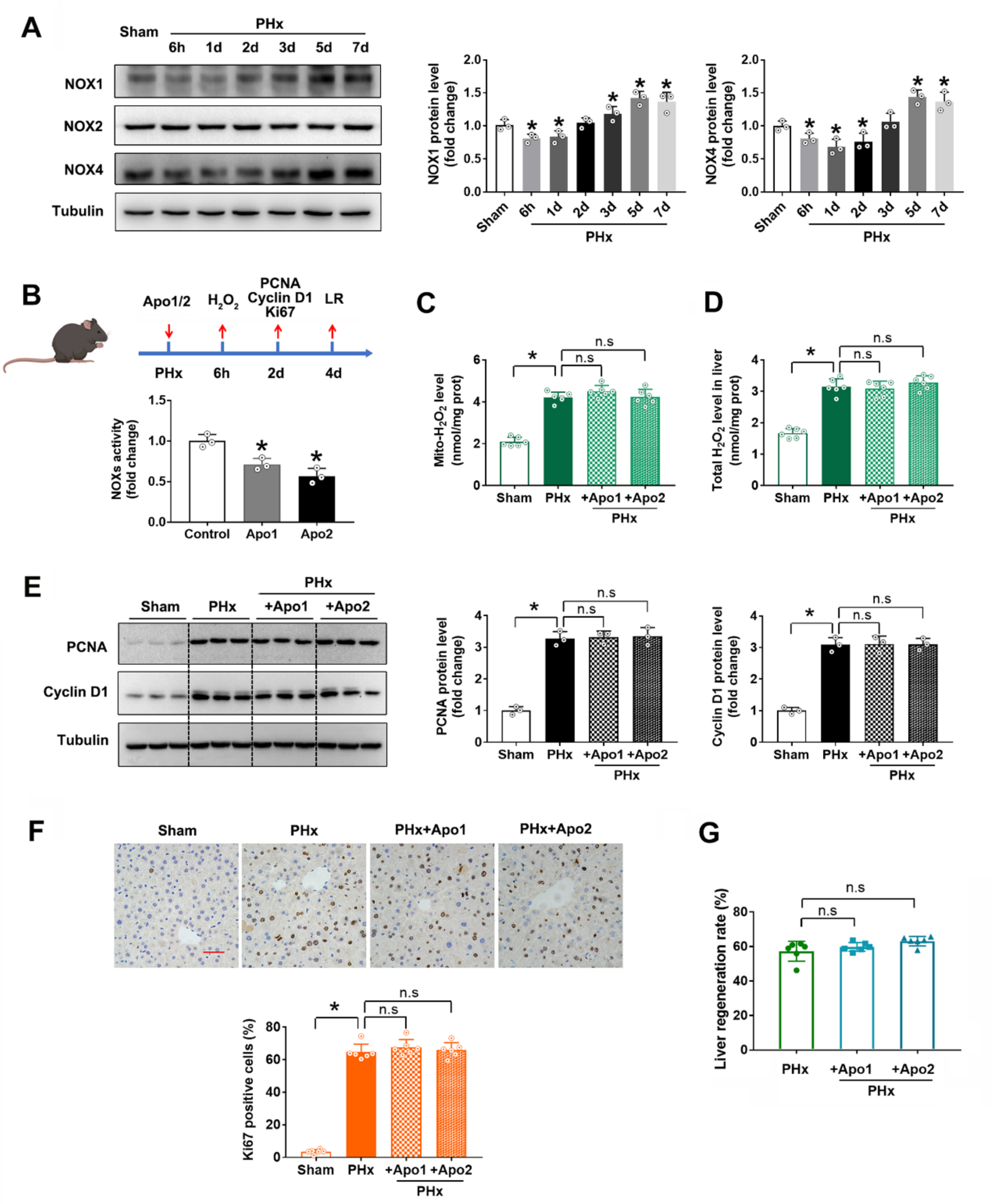

S-Fig 4: NOXs were not involved in the elevated H_2O_2 production and LR after PHx in mice. (A) Western blot analysis and quantification of NOXs protein levels at different time points (6 h, 1 d, 2 d, 3 d, 5 d and 7 d) after the C57 mice were subjected to PHx (three independent experiments). B-F: The mice were intraperitoneal injected

with NOXs inhibitor Apocynin (5mg/kg BW for Apo1 and 50mg/kg BW for Apo2) immediately after PHx and followed by once every day. The H_2O_2 production was detected at 6 hours and LR was determined at the 2th day after PHx. (B) Schematic representation of the experimental procedure and the NOXs activities in Apocynin-treated normal mice, n=3. (C) and (D) Mitochondrial H_2O_2 and total H_2O_2 detected by Amplex red kit, n=6. (E) Western blot analysis and quantification of PCNA and Cyclin D1 (three independent experiments). (F) Immunohistochemistry staining and quantification of Ki67, bar = 50 μm, n=6. (G) LR rate, n=6. Data are shown as means ± SEM. *$p < 0.05$ vs Sham group.

S-Fig 5

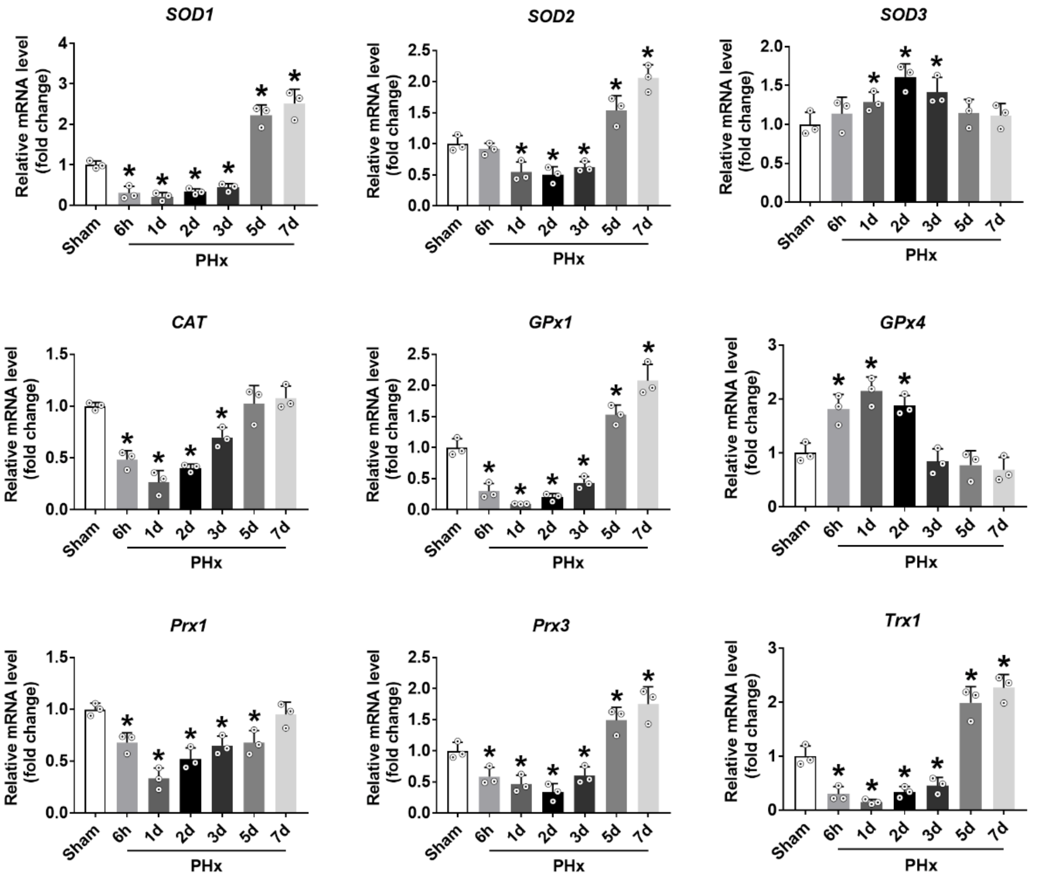

S-Fig 5: The expression of antioxidants at different time points after PHx in mice. After the C57 mice were subjected to PHx, the expression of antioxidants in the liver was determined at different time points (6 h, 1 d, 2 d, 3 d, 5 d and 7 d) by Q-PCR analysis. Data are shown as means ± SEM of three independent experiments. *$p < 0.05$ vs Sham group.

S-Fig 6

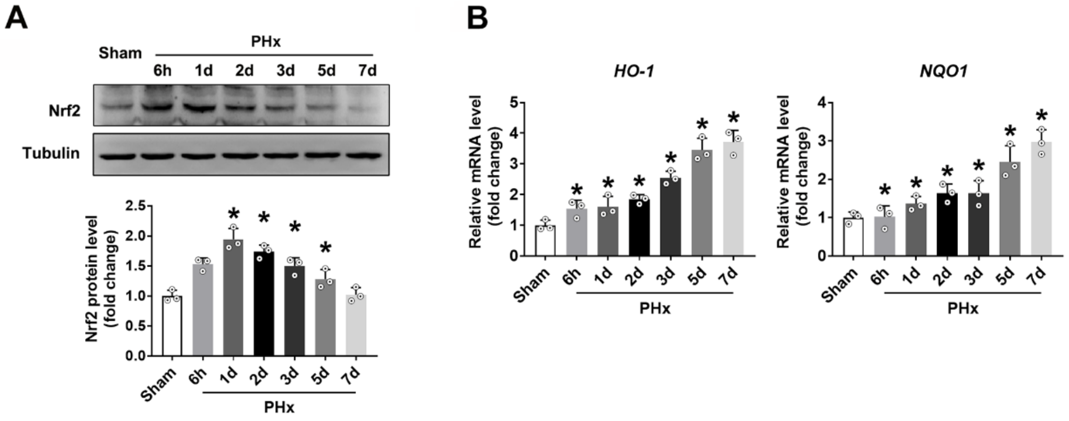

S-Fig 6: The protein level of Nrf2 and the expressions of its target genes (HO-1 and NQO1) in the downstream at different time points after PHx in mice. After the C57 mice were subjected to PHx, the protein level of Nrf2 and the mRNA levels of HO-1 and NQO1 were determined at different time points (6 h, 1 d, 2 d, 3 d, 5 d and 7 d). (A) Western blot analysis and quantification of Nrf2 protein level. (B) Q-PCR determination of mRNA levels of HO-1 and NQO1. Data are shown as means ± SEM of three independent experiments. *$p < 0.05$ vs Sham group.

S-Fig 7

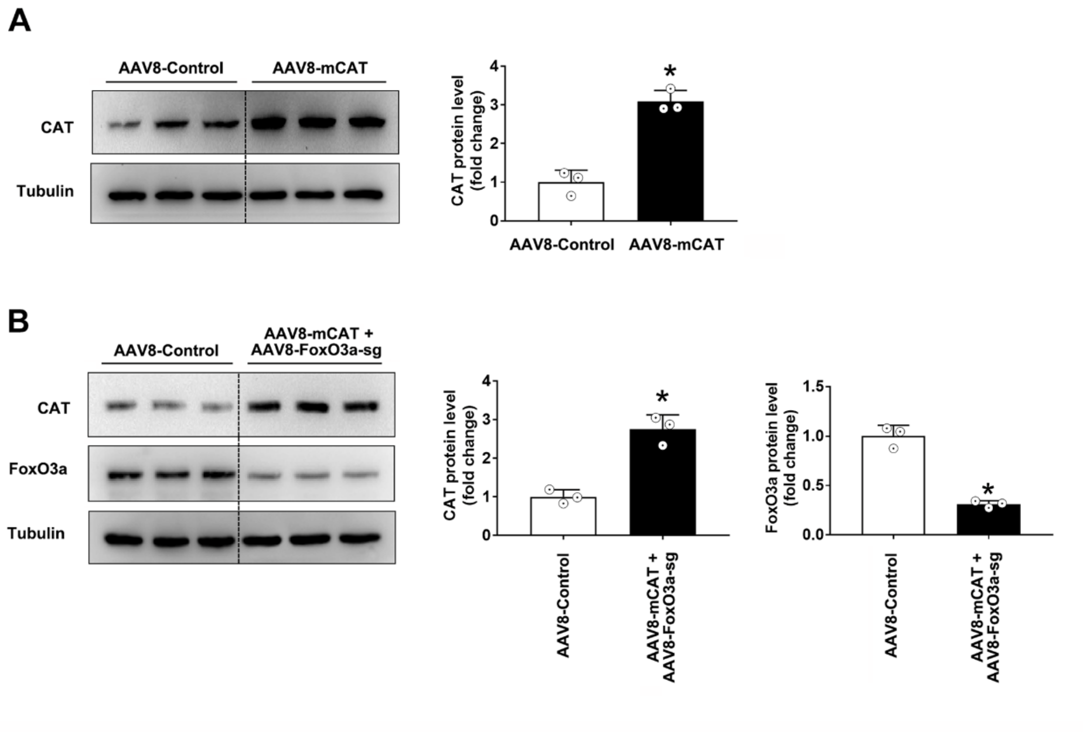

S-Fig 7: The mice with overexpression of mCAT or knockdown of FoxO3a specifically in the liver were generated by AAV8 mediated gene overexpression or Crispr-Cas9 technology. (A) Western blot analysis and quantification of CAT protein level in the liver of mice injected with a recombinant AAV8 carrying CAT gene with mitochondria targeted sequence, n=3. (B) Western blot analysis and quantification of CAT and FoxO3a protein levels in the liver of mice injected with a recombinant AAV8 carrying CAT gene with mitochondria targeted sequence, and a recombinant AAV8 carrying the sgRNA sequence for FoxO3a, n=3. Data are shown as means ± SEM. *$p < 0.05$ vs Control.

S-Fig 8

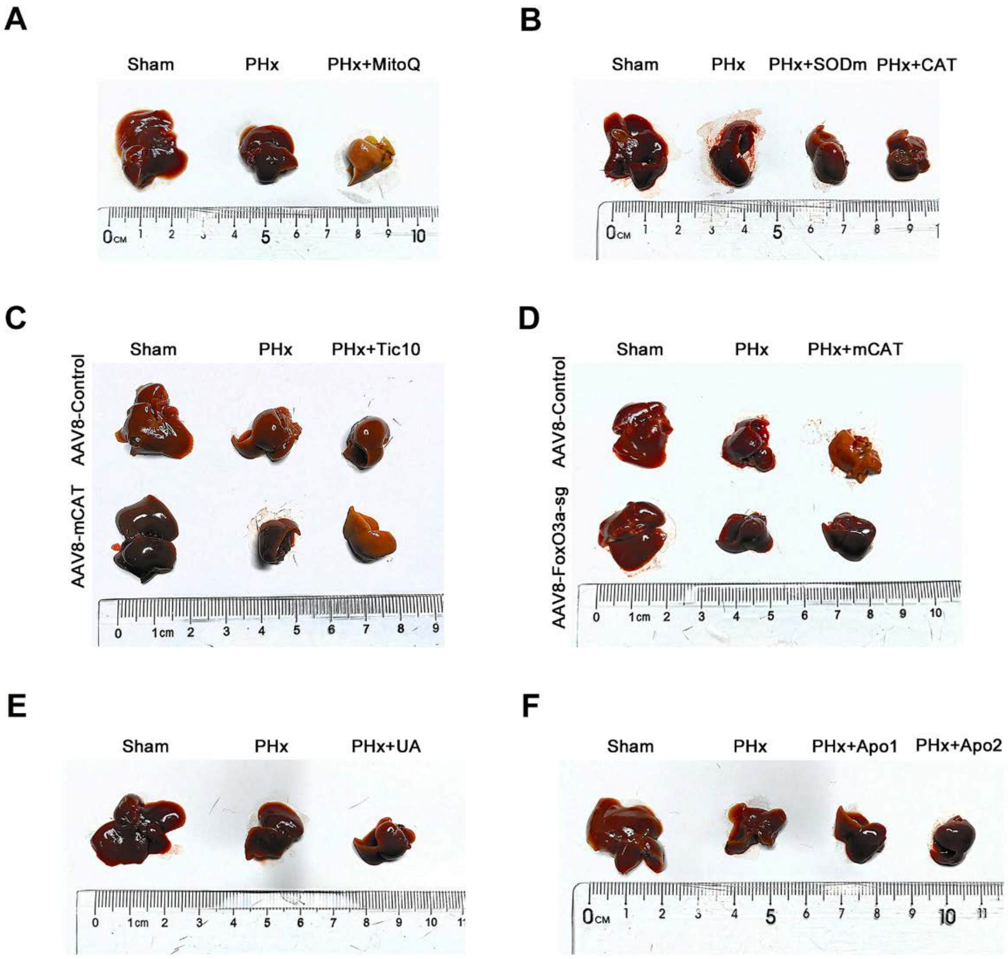

S-Fig 8: The macroscopic images of remnant liver in the all experiments of the present study.

S-Table 1: Antibodies used in this study.

Antibody	Company	CatNo	Dilution
PCNA	Abcam	ab92552	1:1000
Cyclin D1	Abcam	ab134175	1:1000
CAT	Abcam	ab76110	1:1000
NOX1	Abcam	ab131088	1:1000
NOX2	Abcam	ab129068	1:1000
NOX4	Abcam	ab133303	1:1000
Lamin B1	Abcam	ab133741	1:1000
FoxO3a	Cell Signaling Technology	#2497	1:1000
p-FoxO3a-Ser253	Cell Signaling Technology	#13129	1:1000
p-FoxO3a-Ser294	Cell Signaling Technology	#5538	1:1000
Akt	Cell Signaling Technology	#4691	1:1000
p-Akt	Cell Signaling Technology	#4060	1:1000
Erk	Cell Signaling Technology	#4695	1:1000
p-Erk	Cell Signaling Technology	#9101	1:1000
p27	Cell Signaling Technology	#3698	1:1000
Parkin	Cell Signaling Technology	#4211	1:1000
Nix	Cell Signaling Technology	#12396	1:1000
Ki67	Servicebio Technology	GB111499	1:500
PINK1	Santa Cruz Biotechnology	sc-517353	1:1000
FUNDC1	Bioss Technology	bs-13227R	1:500
Tubulin	Bioworld Technology	BS1699	1:5000

S-Table 2: Primers used in this study.

Gene name	Primer sequences
p27	Forward: 5`-GGACCAAATGCCTGACTCGT-3`<br>Reverse: 5`-CGCTTCCTCATCCCTGGAC-3`
SOD1	Forward: 5`-GAGACCTGGGCAATGTGACT-3`<br>Reverse: 5`-GCCAATGATGGAATGCTCTC-3`
SOD2	Forward: 5`-CGGCCTACGTGAACAATCTC-3`<br>Reverse: 5`-CCAGAGCCTCGTGGTACTTC-3`
SOD3	Forward: 5`-CATGTCAAATCCAGGGGAGT-3`<br>Reverse: 5`-GGTCAAGCCTGTCTGCTAGG-3`
CAT	Forward: 5`-ACCAGGGCATCAAAAACTTG-3`<br>Reverse: 5`-GCCATAATCCGGATCTTCCT-3`
GPx1	Forward: 5`-GGTTCGAGCCCAATTTTACA-3`<br>Reverse: 5`-CATTCCGCAGGAAGGTAAAG-3`
GPx4	Forward: 5`-CCCATTCCTGAACCTTTCAA-3`<br>Reverse: 5`-CAGTACTGGGGAGGCTCTTG-3`
Prx1	Forward: 5`-CACGGAGATCATTGCTTTCA-3`<br>Reverse: 5`-CCAATCACTTGGCAGTTGAG-3`
Prx3	Forward: 5`-TCGTCAAGCACCTGAGTGTC-3`<br>Reverse: 5`-CTGGAACGCCTTTACCAAAC-3`
Trx1	Forward: 5`- TGTCGTGGTGGACTTCTCTG -3`<br>Reverse: 5`- TGATCATTTTGCAAGGTCCA -3`
HO-1	Forward: 5`-TGCTCGAATGAACACTCTGG-3`<br>Reverse: 5`-AAGGCGGTCTTAGCCTCTTC-3`
GCLC	Forward: 5`-AATGACAGTTGCCAGATGGA-3`<br>Reverse: 5`-AGTCAGGATGGTTTGCGATAA-3`
GCLM	Forward: 5`-AGTTGCACAGCTGGACTCTG-3`<br>Reverse: 5`-AGGCTGTAAATGCTCCAAGG-3`
NQO-1	Forward: 5`-CAGATCCTGGAAGGATGGAA-3`<br>Reverse: 5`-GGCTGCTTGGAGCAAAATAG-3`
β-actin	Forward: 5`-TGTTACCAACTGGGACGACA-3`<br>Reverse: 5`-GGGGTGTTGAAGGTCTCAAA-3`
