# Supplementary material for: Mitochondria-derived H2O2 triggers liver regeneration via FoxO3a signaling pathway after partial hepatectomy in mice
Source: Cell Death Dis. 2023 Mar 28;14(3):216. doi: 10.1038/s41419-023-05744-w (PMC10050396; doi:10.1038/s41419-023-05744-w)

Fig 1D

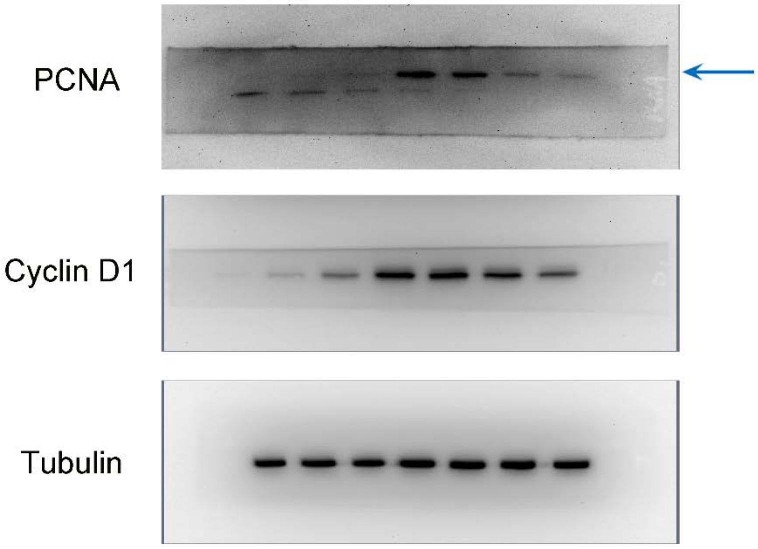

Fig 2B

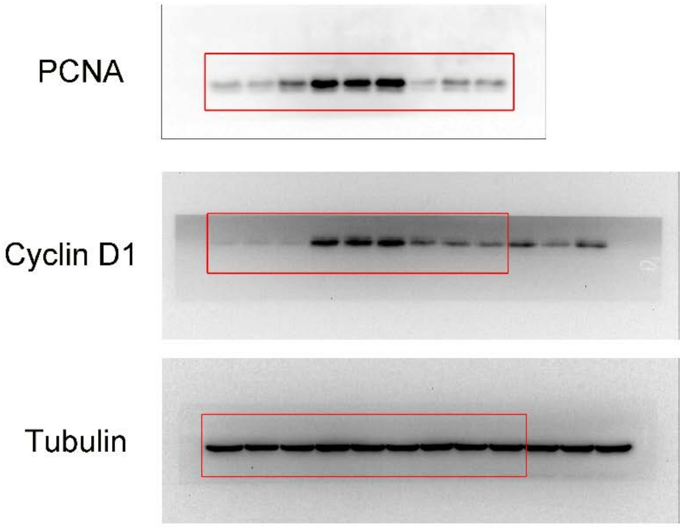

Fig 3F

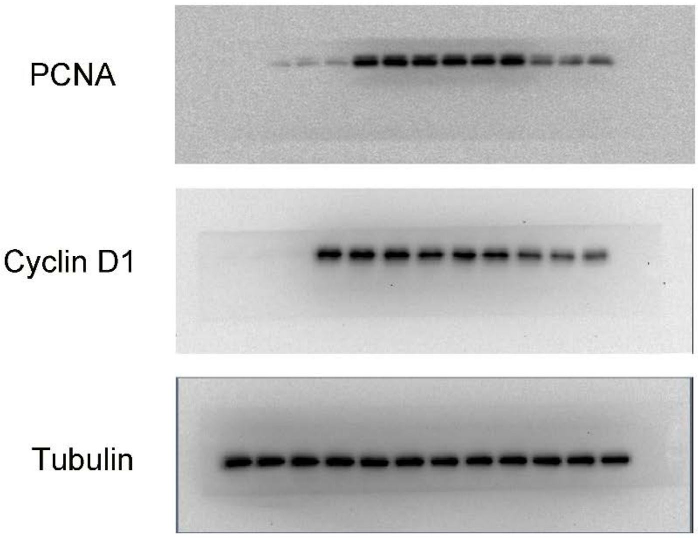
PCNA
Cyclin D1
Tubulin

Fig 4A

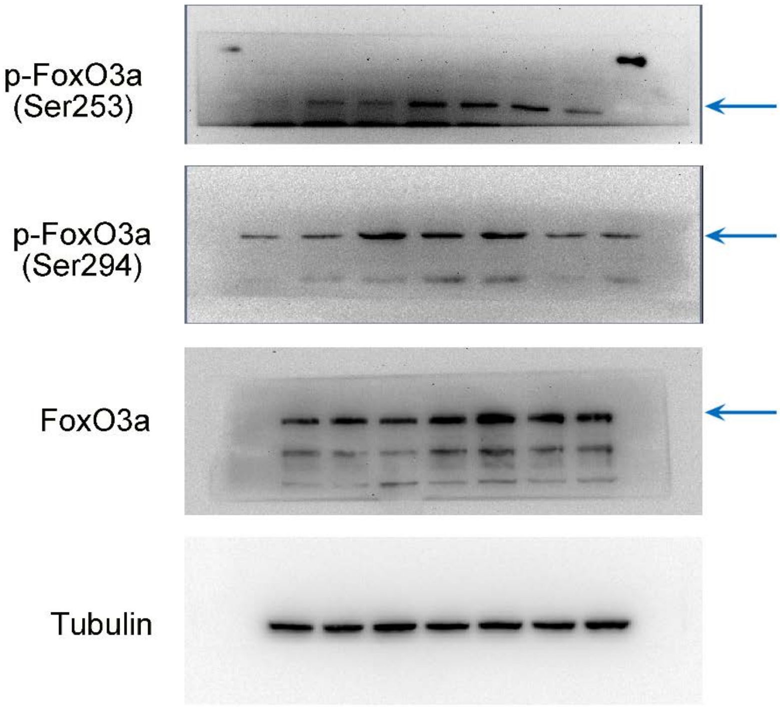
p-FoxO3a
(Ser253)
p-FoxO3a
(Ser294)
FoxO3a
Tubulin

Fig 4B

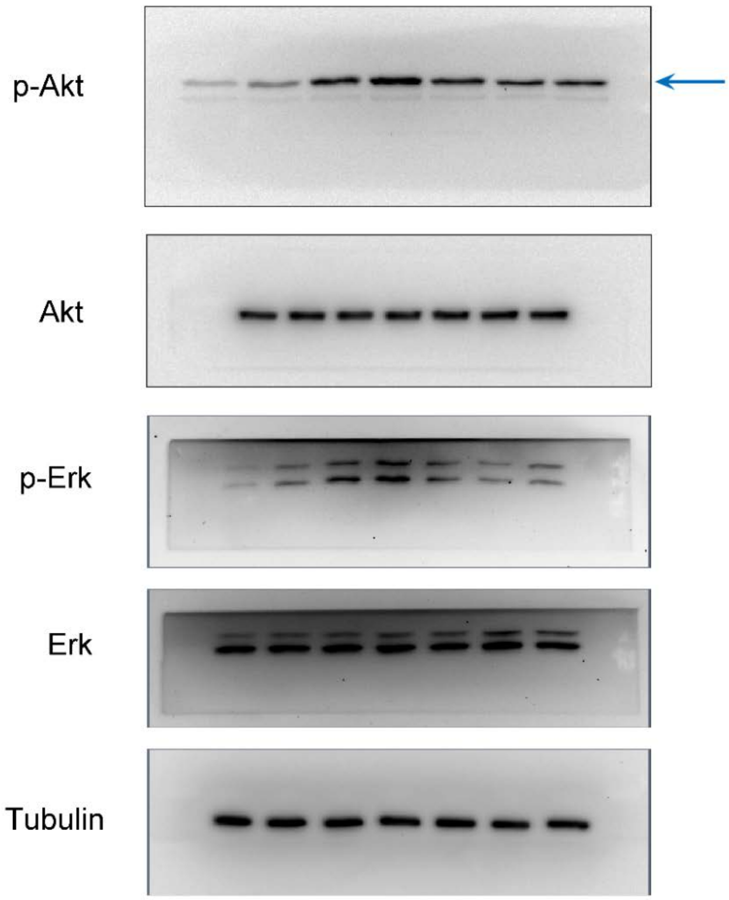

Fig 4C

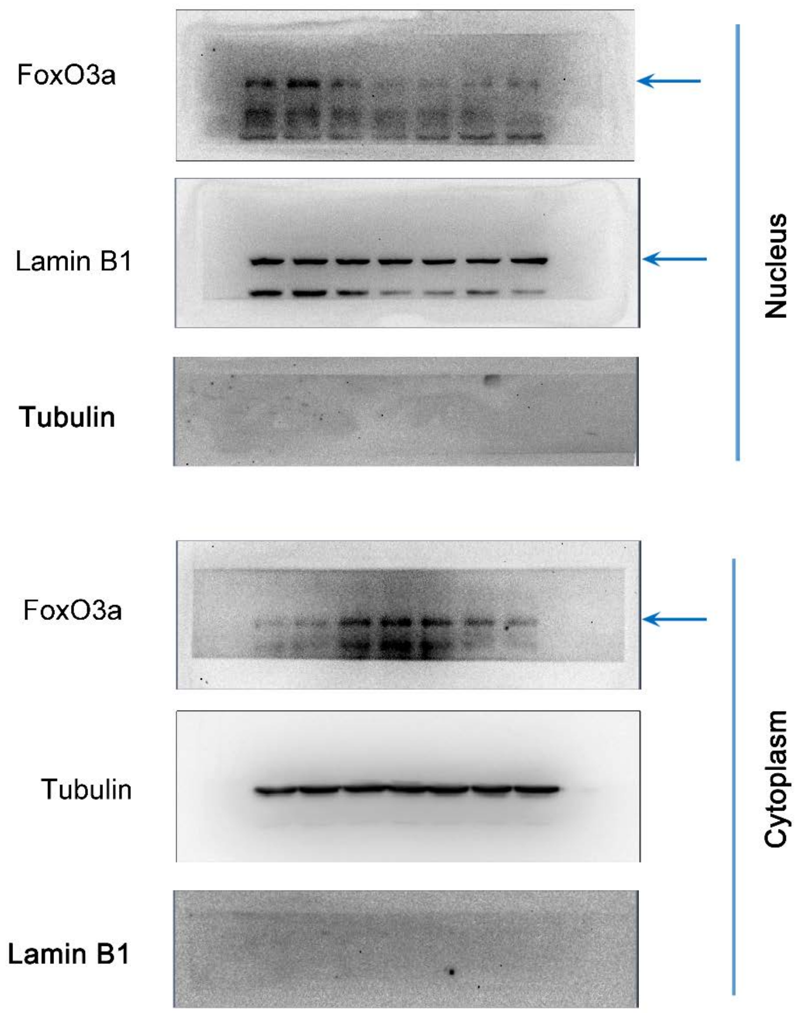
FoxO3a
Lamin B1
Tubulin
Nucleus
FoxO3a
Tubulin
Lamin B1
Cytoplasm

Fig 4E

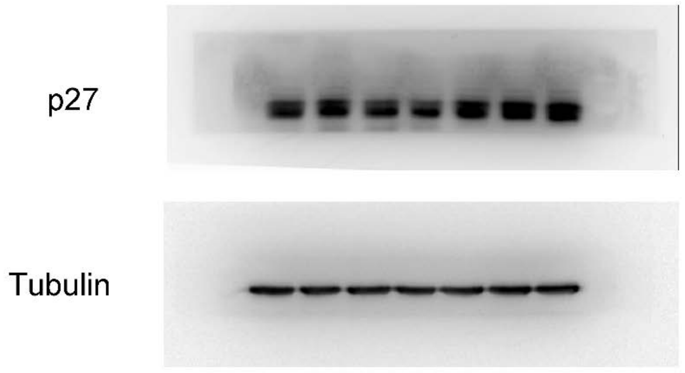
p27
Tubulin

Fig 5A

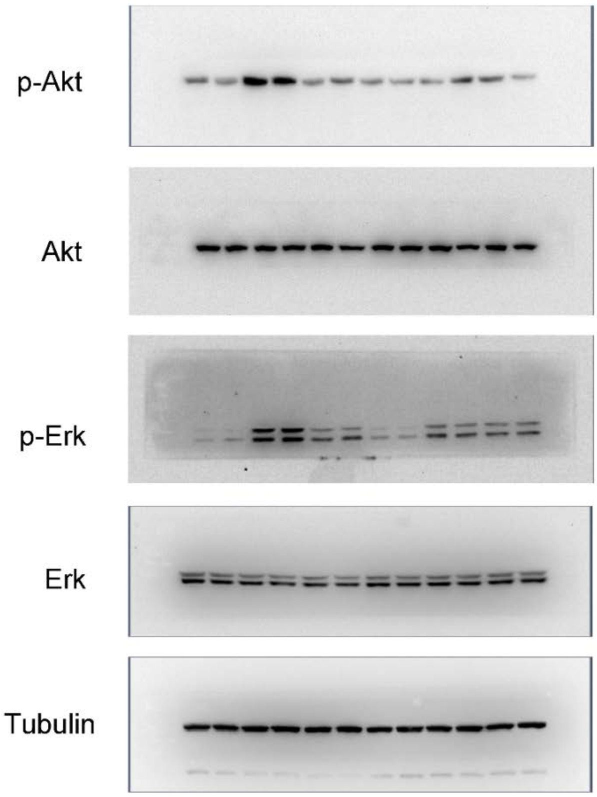

p-Akt
Akt
p-Erk
Erk
Tubulin

Fig 5B

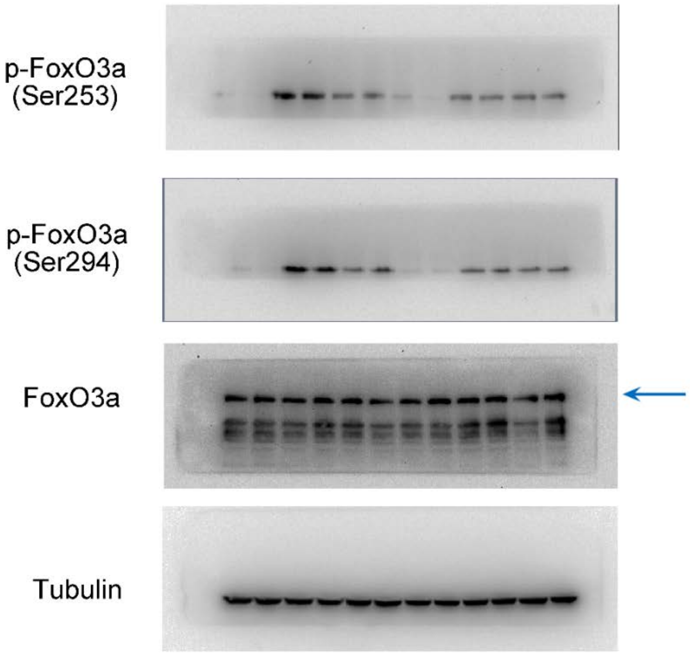

p-FoxO3a
(Ser253)
p-FoxO3a
(Ser294)
FoxO3a
Tubulin

Fig 5C

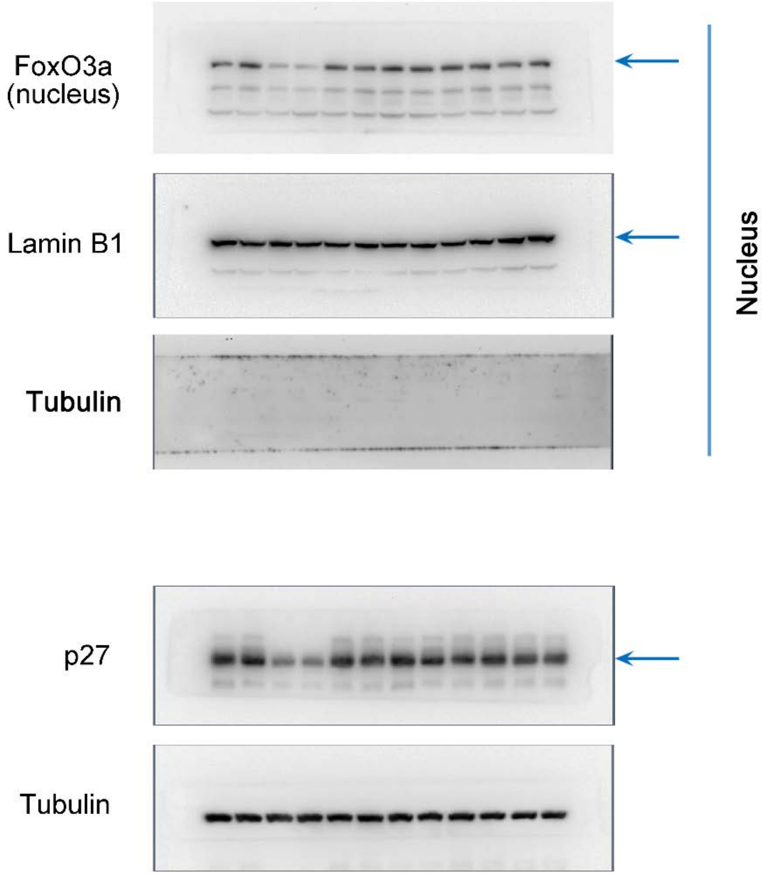
FoxO3a
(nucleus)
Lamin B1
Tubulin
Nucleus
p27
Tubulin

Fig 5D

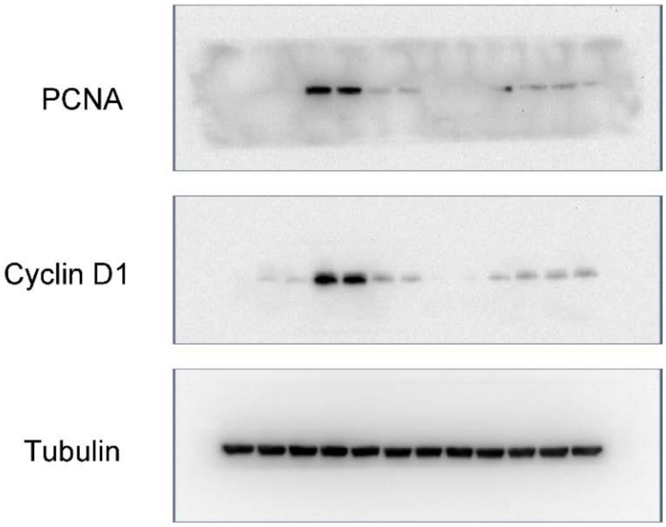
PCNA
Cyclin D1
Tubulin

Fig 6A

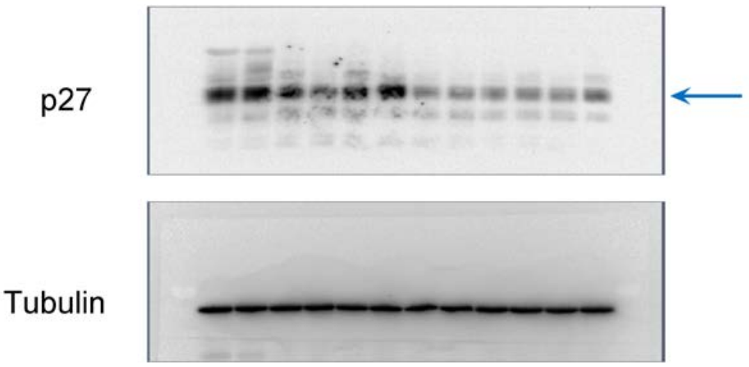

Fig 6B

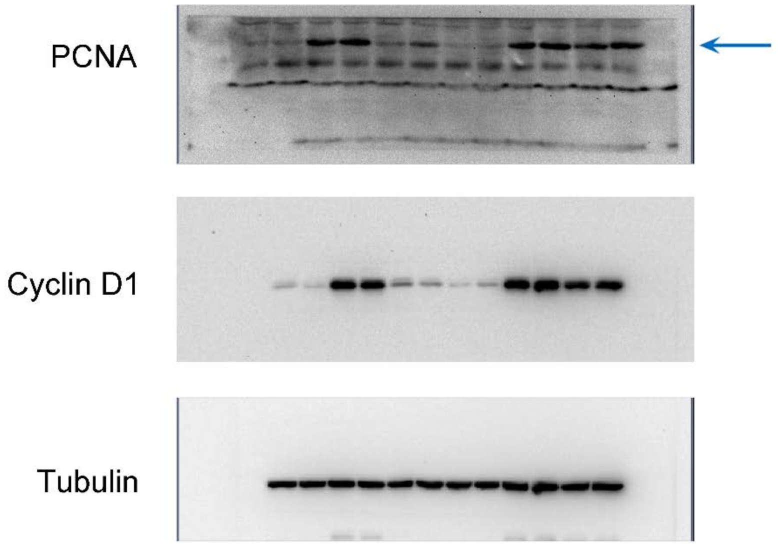

S-Fig 2

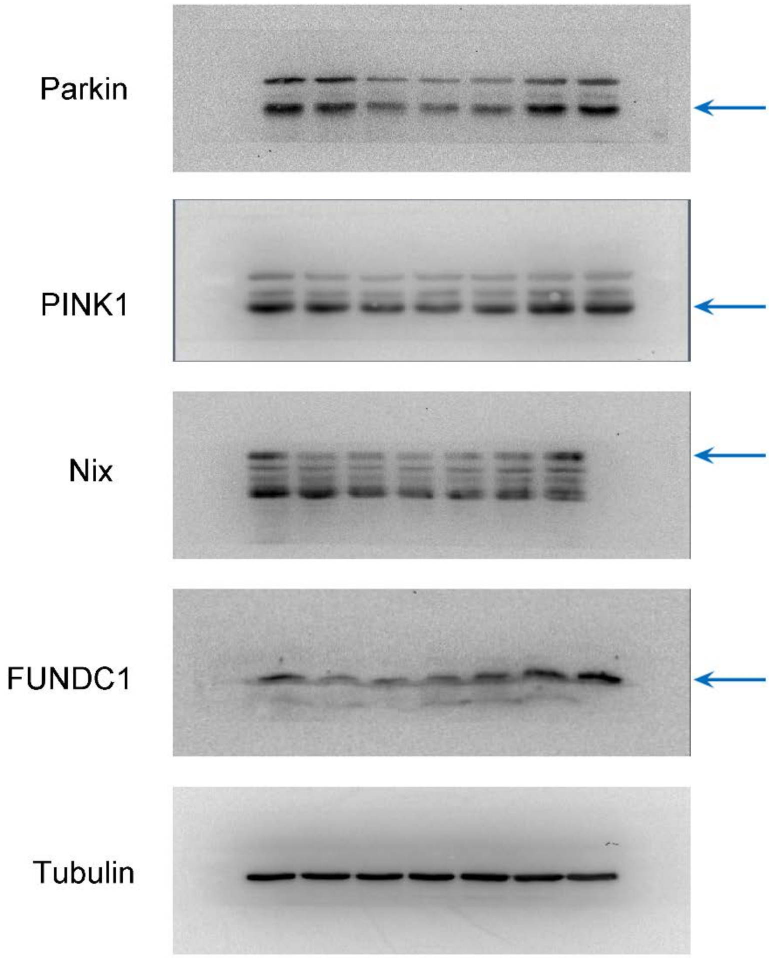

S-Fig 3A

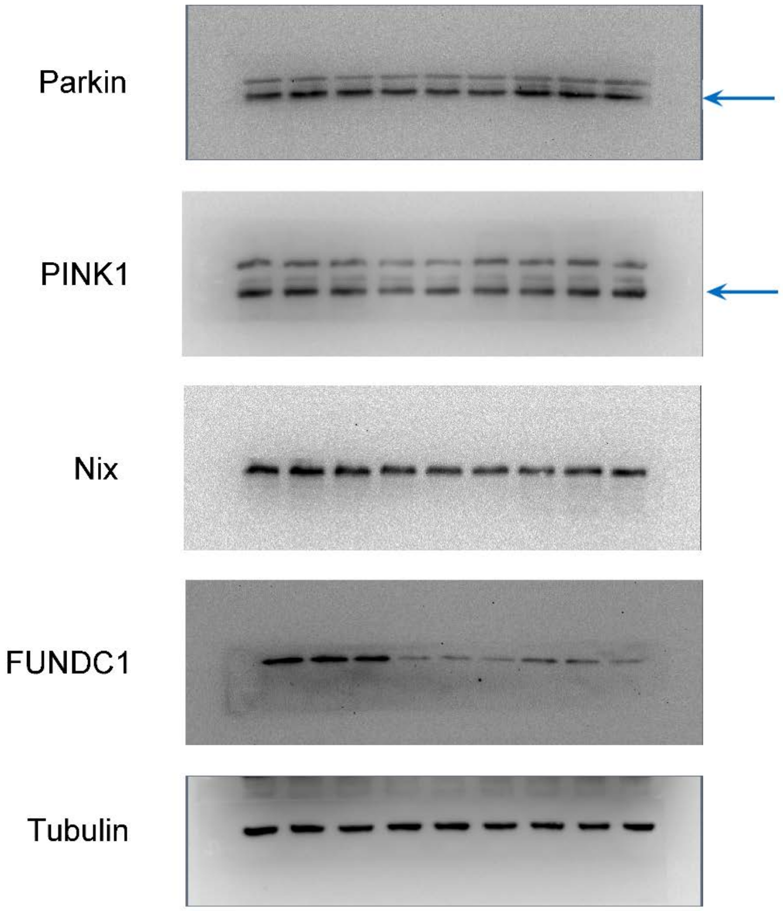

S-Fig 3G

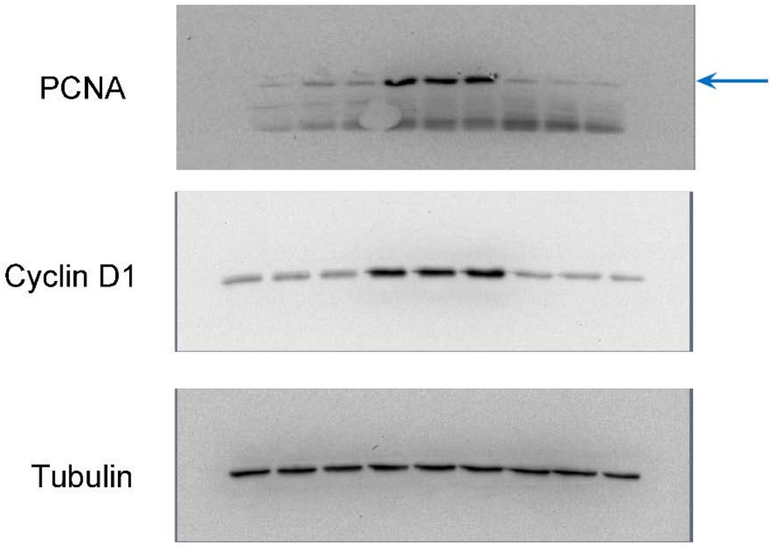

S-Fig 4A

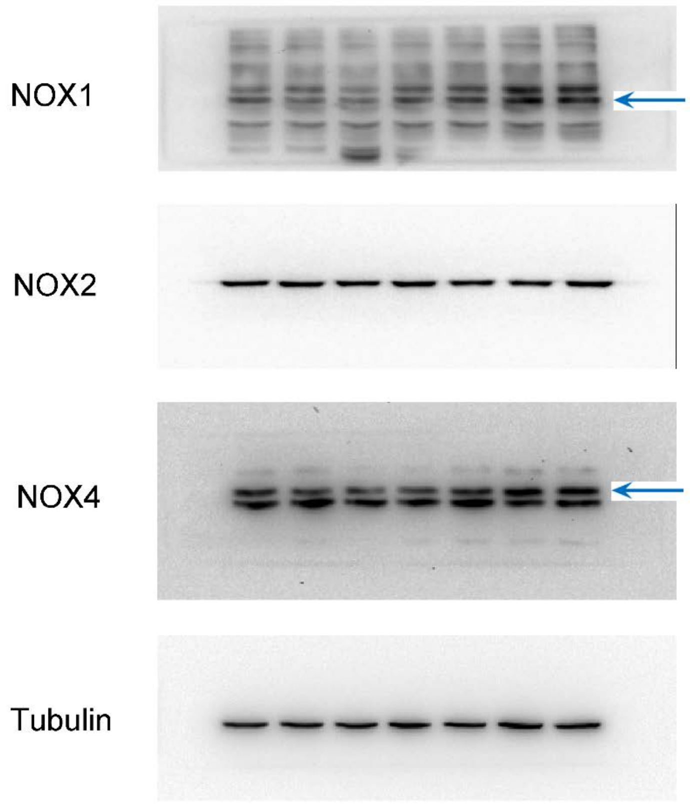

S-Fig 4E

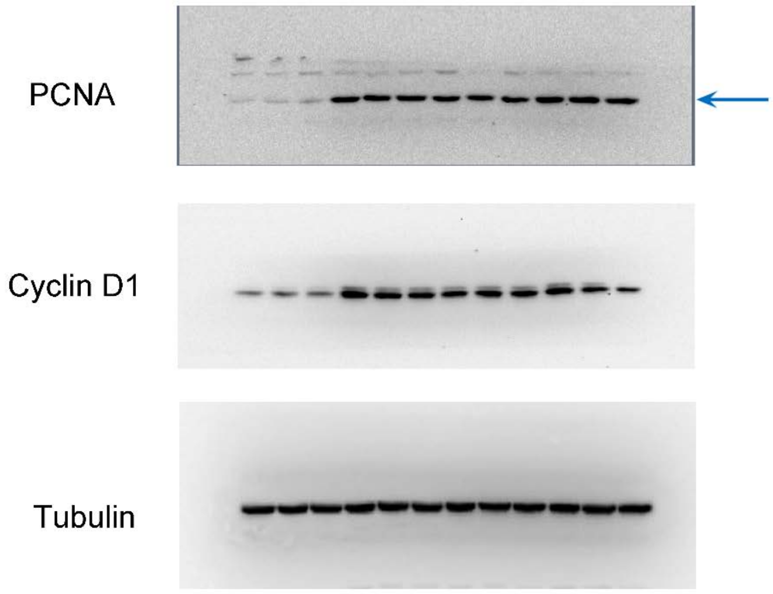

S-Fig 6

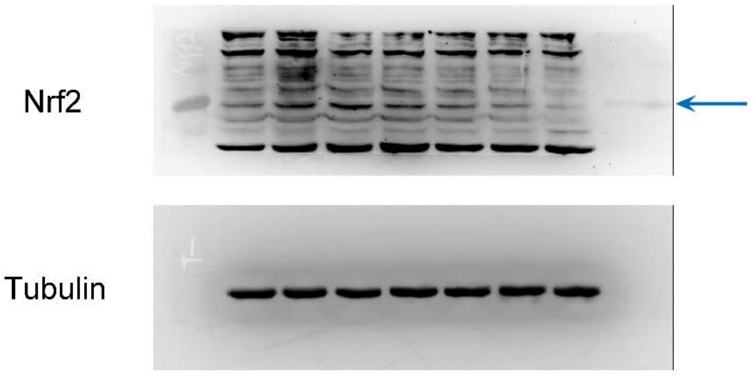

S-Fig 7A

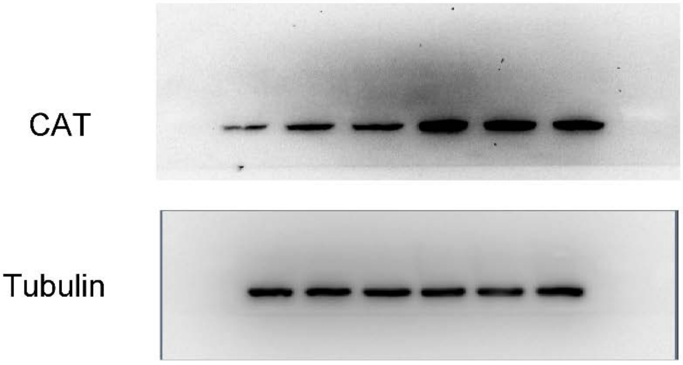

S-Fig 7B

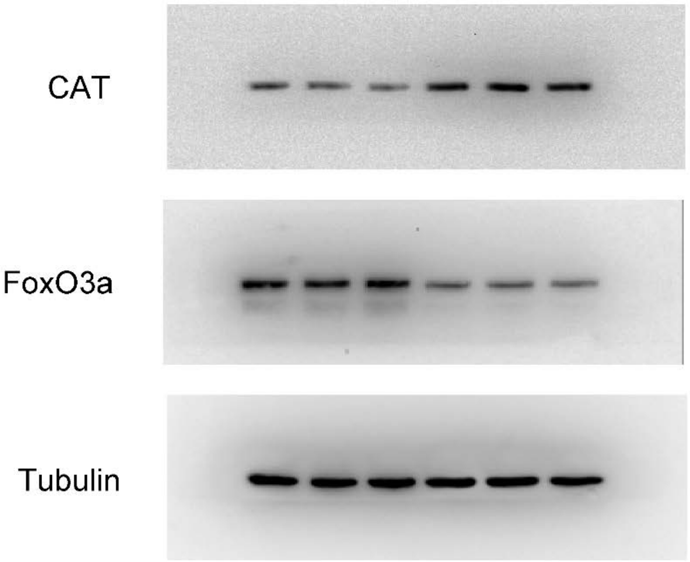

Supplement: Supplementary file 2 — Original Data File [file 41419_2023_5744_MOESM2_ESM.pdf]
